# Overconfidence Among Young Decision-Makers: Assessing the Effectiveness of a Video Intervention and the Role of Gender, Age, Feedback, and Repetition

**DOI:** 10.1038/s41598-020-61078-z

**Published:** 2020-03-04

**Authors:** Dominik M. Piehlmaier

**Affiliations:** 1 School of Human Ecology, University of Wisconsin-Madison, 1300 Linden Dr., Madison, WI 53706 USA; 2 Present Address: University of Sussex Business School, Jubilee Building, 9SL Brighton, UK

**Keywords:** Psychology, Human behaviour

## Abstract

Child development research on overconfidence suggests that the bias is present and persistent in preschoolers and kindergartners. However, little is known about what drives overconfidence among young decision-makers, how it changes over a large number of repetitions, and whether such changes differ by gender or age. The current experimental study analyzes data from 60 children, aged 4 years 0 months to 6 years 10 months, who played 60 turns of the Children’s Gambling Task and provided regular estimates on their performance. A video intervention, designed to demonstrate the consequences of disadvantageous choices, was tested in a double-blind randomized controlled trial to assess its impact on overconfidence. The results show that every third participant remained overconfident even after 60 trials and constant feedback. Unlike previously reported, gender seems to be a determining factor in this process. Lastly, providing additional information through a video intervention appears to have no impact on participants’ overconfidence levels.

## Introduction

Overconfidence describes the tendency to overestimate one’s skills and underestimate the impact of risk and ambiguity on a given outcome^1^. The presence and influence of overconfidence on young decision-makers (4 years 0 months and older) has been consistently shown in a multitude of empirical studies over the course of several decades. The majority of these studies relied on memory tasks that compared children’s predicted performance to their actual, observed outcome (e.g., performance on a picture recall task)^2–4^. In all of these studies, the average participant significantly overestimated their own performance which resulted in a substantial discrepancy between prediction and actual recall. Additionally, there is evidence that overconfidence in early childhood and primary education slightly decreases with age^3,5,6^. For instance, preschoolers and kindergartners seem to be more likely to overstate their recall performance compared to third-graders. A possible explanation for this finding is superior metacognitive abilities among older children who have a greater capacity to accurately recall and evaluate their past performance and assess their own predictions^7^.

Younger decision-makers also exhibit more persistence in excessively confident predictions, even after repeating the task, recalling their past performance before providing an estimate for the repetition, or assessing the performance of another child^8^. The former aspect suggests that children do not develop underconfidence with practice (UWP) at an early age. UWP describes the propensity to initially observe excessively confident behavior which shifts to underconfidence after a sufficiently large number of repetitions. However, the approximate age range may be disputable, as Finn and Metcalfe^9^ found that even fifth-graders at a mean age of 10 years 0 months showed consistent overconfidence, while Lipko and colleagues^5^ concluded that third- (mean age: 8 years 11 months) but not first-graders (mean age: 6; 11) exhibited UWP.

It may be noted that the majority of the aforementioned studies did not report or find any discrepancy between the performance or self-assessment of girls and boys in their samples^3–6,8,9^. This stands in stark contrast to empirical work assessing overconfidence among adults that commonly find a gender effect^10–12^. Even if an effect among young decision-makers was detected, it might have been deemed spurious or described as “[not] *very credible, interpretable, or interesting*”^2^ (p. 334). That being said, Pressley and Ghatala^7^ found that girls in first and second grade can be associated with a more precise assessment of their performance compared to their male counterparts. It is unclear whether these deviating findings are sample-specific or arise from the difference in the underlying procedures (i.e., memory task in the former studies and a picture vocabulary test in the latter).

Furthermore, few studies used randomized controlled designs to identify mechanisms underlying age-related changes in overconfidence. These studies provide conflicting evidence with some suggesting that interventions designed to engage metamemory processes (i.e., reflection and knowledge about one’s memory) can reduce the bias. For instance, Stipek *et al*.^13^ played a game of deception with sixty 4-year-olds. Participants were asked to lift a metal ball up a tower on a cart that was secretly controlled by magnetic force. The preschoolers were randomly allocated into experimental arms that either had to make prediction about their own performance or about the performance of a gender-matched child who played the same game on TV. Both arms included three groups receiving incentives in the form of marbles, explicit feedback regarding their past “performance” (which could not be influenced by the participants), or no intervention (i.e., control condition). If incentivized, mean predictions between self and others did not differ. However, if participants did not receive any intervention, their own future performance was perceived to be superior to the outcome of the other child. The reverse is true when preschoolers were explicitly reminded about their past performance. Yet, small intragroup sample sizes of 10 participants and a notable absence of influence on the actual outcome, diminish the generalizability of the findings. Powel and colleagues^14^ asked 22 children (aged 4; 2–6; 9) to provide confidence estimates about their ability to throw 10 beanbags into a basket. All participants showed overconfidence in their throwing skills, but accuracy seems to improve with age. Lastly, Lagatutta and Sayfan^15^ used a narrative of positive, negative, or ambiguous events to assess attitude and predictions of 265 children (mean age 4; 6–8; 11) and adults (mean age 20; 0). Contrary to previous studies, the results suggested that unjustifiable confidence in past events with limited information value increased with age. A possible explanation for these conflicting results may be found in the underlying experimental tasks. The former studies that reported a decrease of the bias with age utilize physically executable tasks (e.g., lifting a metal ball, throwing beanbags, etc.) while the latter work relied on an abstract assessment of a future event that required a judgment under uncertainty without any physically executable actions. Prior research suggests that overconfidence is domain specific which might explain why experimental tasks that require different cognitive processes lead to competing implications regarding the role of age^16^.

Despite the aforementioned evidence that incentives and explicit feedback influences overconfidence, none of the aforementioned studies consistently incorporated these aspects into their experimental designs^13^. Incentives have limited impact if and only if intrinsic motivation to perform and calibrate one’s decisions is sufficiently high. For instance, children may be inclined to purely guess how many pictures they can memorize in the absence of a performance-based incentive structure. This outcome may be significantly different to their actual potential to memorize details^17^. Furthermore, studies of metamemory heavily rely on the difference between prediction and skill-based performance. However, overconfidence is most prevalent and arguably more problematic in real-life situations that are characterized by a notable degree of uncertainty^18^. That is, overconfidence is a function of excessive optimism, self-serving traits, and risk or ambiguity. The latter aspects are all but absent in metamemory tasks. It may well be that the findings from the aforementioned studies are largely driven by the novelty of the experimental tasks and the underlying lack of knowledge of children on how to assess their skills for the given settings. A large number of repetitions could lead to more calibrated estimations.

The current study incorporates all of these aspects. The experiment is based on the Children’s Gambling Task (CGT) which is an age-appropriate adaption of the Iowa Gambling Task that was developed to examine hot executive functions among 3- to 4-year-olds^19^. The card game includes elements of skill and luck which mimics a situation under uncertainty more closely than memory tasks. In addition, each participant plays 60 turns which account for the novelty of the game and offer ample opportunity to practice. Actual performance is incentivized by stickers in the value of the drawn cards (henceforth, payoffs). The game is played in 10-turn blocks, after which children are asked to estimate their performance on the next 10-turn block. These estimates provide an explicit feedback regarding past performance as each participant is reminded of the current number of stickers to play with (i.e., payoff balance). After 30 turns, half of the participants receive an experimental intervention consisting of a video showing a gender-matched cartoon child who plays the same game but loses all stickers after several turns. The control group is exposed to an unrelated card game instruction (“Old Maid”) of approximately equal length and design.

One aim of this study was to test overconfidence as well as potential gender and age effects among preschoolers and kindergartners in a situation of uncertainty. A second aim was to test whether a simple reminder of an inferior game strategy could impact the calibration of estimates of young decision-makers. In consideration of the aforementioned evidence, it was hypothesized that the intervention group would exhibit more calibrated assessments of their future performance compared to controls. Estimates for the 30 trials prior to the intervention should not substantially differ between the two groups. Given the array of prior studies that did not find a gender effect, it is hypothesized that girls and boys are equally miscalibrated. Lastly, it is hypothesized that overconfidence decreases with age due to the aforementioned abundance of empirical evidence. The following section describes the sample, the experimental task, and the procedure in detail.

## Method

### Participants

Sixty participants (48.33% females; female mean age: 4 years 9 months; male mean age: 4 years 10 months; general age range: 4 years 0 months to 6 years 10 months, mean 4 years 10 months) and their caregivers were recruited at on-campus preschool facilities (60%) and a children’s museum in a Midwestern city between fall 2017 and fall 2018. Parents provided informed consent and preschoolers verbally assented. One female preschooler did not assent, and another child withdrew her assent before the game started; due to ethical reasons, the two participants were excluded from further consideration. Consequently, there are 58 valid responses-29 in each experimental arm. The response rate for all eligible preschoolers was at 70.37% and the subsequent participation rate at 78.94%. The only actively enforced inclusion criterium was age. The targeted age range is in accordance with prior research using metamemory or general metacognitive tasks^6,15,17^. Preschoolers were drawn from a population with 98.20% married or live-in partner parents, 55.36% had four people in their household and 69.65% had an annual household income of $100.000 or more (8.93% did not answer). The vast majority of parents was between 30–49 years of age (88.28%), well-educated (72% Master’s or higher), and non-Hispanic (90%) who self-identified as 66.77% white, 17.12% Asian, and 9% black (7.21% did not answer). Based on previous studies at the children’s museum, 82% identify as white and non-Hispanic with an average education of 17.12 years (i.e., more than bachelor degree) and a mean MacArthur Scale of Subjective Social Status of 7.85^20^. Institutional requirements preconditioned that all participants were fluent in English and did not have a history of diagnosed disorders. In summer 2017, a pretest (n = 4) was conducted to assess the appropriateness of the experimental design; no substantial changes were made. The study was initially approved by the Education and Social/Behavioral Science Institutional Review Board at the University of Wisconsin-Madison in February 2017 (ID 2016-1565-CP001) and periodically reapproved in January 2018 and December 2019. All methods were performed in accordance with relevant guidelines and regulations.

### Task

**Figure 1:**
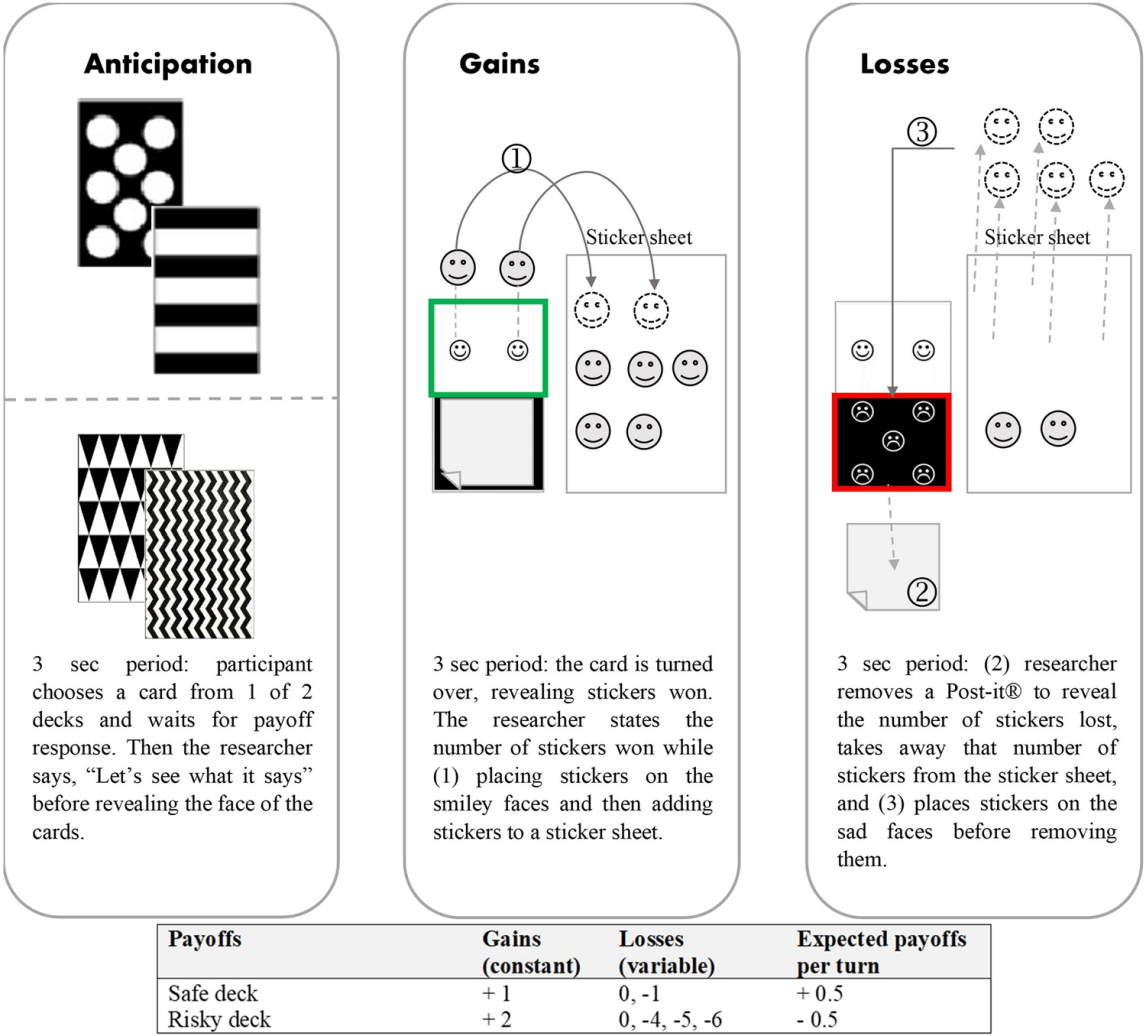
Game Setup. Adapted from Faja *et al*.^22^ (License Number 4744400415515).

The experimental task was an age-appropriate adaption of the Iowa Gambling Task. The original game consists of four decks with different payoff structures; two of those decks have superior payoffs compared to the remaining two. Adult controls without diagnosed prefrontal damages or decision-making deficiencies quickly deviate from picking the more risky and inferior decks and select the safer and more advantageous ones^21^. The preschool-friendly adaptation of the card game, called the Children’s Gambling Task (CGT), has been tested with children as young as 3 years 1 month of age and consists of two decks, one of which has superior average payoffs compared to the other (see Fig. 1)^19^. Despite the fact that the general setup of the game followed previous studies (e.g., the order in which cards were presented; ibid., p. 151) in order to replicate the main findings, there were several notable changes. Incentives in the form of candy were replaced by stickers. This increases the participation rate by minimizing the risk of exclusion due to dietary restrictions and parental disapproval. Stickers have been used as an incentive option in a previous experiment^22^. In line with Kerr and Zelazo^19^, a turn was defined as a child’s selection of a card from one of the two decks and turns were broken into blocks of 10. However, in this experiment, all participants were paused and asked to provide an estimate of their future performance for the next 10 cards based on the preceding block (Q: “*Now, how much do you think you will win with the next 10 cards?*”, A: “*More*”, “*About the same*”, “*Less*”, “*Don’t know*”). The confidence interval was phrased in accordance with the developmental stage of the target group. Children had to decide whether they think they will win more, about the same (coded as +/− 1), or fewer stickers than in the previous block. Participants had the option to state that they do not know what will happen next, though the experimenter did not read it out loud to avoid mental focal points. This essential performance estimate was used for all participants, independent of randomized group allocation, gender, or age. Due to an experimenter error, one boy in the intervention group did not play one of the six blocks; the missing responses could be recovered based on the child’s monotonic selection pattern. The experimenter accidentally continued with the fifth (instead of the fourth) block after showing the video sequence. The child provided all performance estimates and answered all post-intervention comprehension questions. The card selection responses were recovered based on the fact that the participant only chose to play the risky deck in the previous 10 turns prior to the omitted block as well as in all subsequent turns.

### Stimuli

**Figure 2:**
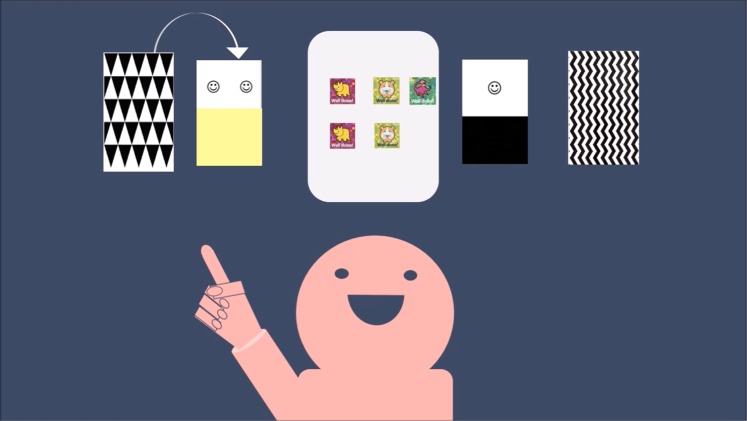
Example of the video sequence for female participants (males in blue). Controls (see Supplementary Material p.19) are presented with a game instruction to “Old Maid”, adapted from Chong^23^. In this example, the clinical group (own illustration) observed Lisa who has five stickers left and has just chosen the risky deck in a randomized situation of triangles to the left and waves to the right.

Participants in the intervention group observed a task-related scenario (Fig. 2); the controls watched a game instruction to the card game “Old Maid” (see Supplementary Material p.19) which is unrelated to the experimental task and lacks the central combination of skill and uncertainty^23^. Furthermore, the voices of narrators and protagonists were generated using deep neural networks to ensure an indistinguishable phonetic experience within and between the experimental arms^24^. Both, narrator and protagonist were gender-matched with the participants. In the video, the patterns on the back of the cards as well as the stickers were identical to a participant’s randomized scenario. Treated participants observed how the protagonist played the CGT. The preschooler in the video initially selected the safe deck once but then constantly picked cards from the disadvantageous deck and lost all stickers within a few rounds. Neither the research nor the narrator criticized the illustrated strategy and the participants were allowed to make their own inferences based on the presented information. In addition, the protagonist was introduced by name (Lisa or Tony) to guarantee that the participants understood their observational role when watching the video.

### Procedure

Trained research assistants, who were unaware of the underlying hypotheses, set up the game in a dedicated room at the child’s preschool or the children’s museum. Participants were familiar with the environment and provided oral assent before the experiment started. One experimenter was responsible for administering the experimental task while another researcher acted as a silent observer. The latter reported all observations in a Qualtrics survey. This online tool was also used to double-blind randomize the group allocation using Qualtrics’ random number generator in order to minimize experimental bias and statistical noise. The randomly chosen pair of decks were placed in the middle of the table in a random order, determined by the same randomization tool; the sticker sheet was visibly located between the two decks. As seen in Fig. 1, there were two pairs of decks with different black-and-white patterns on the back of the cards. The color ratio of the patterns within these pairs was identical. This guaranteed that the design on the back of the cards did not create focal points that would bias the results.

The game started with six practice trials that gave the child the opportunity to get familiar with the setup and to win four stickers to pay for potential future losses. The experimenter reminded the preschooler that all stickers that were left on the sticker sheet at the end of the game could be kept. The child played 30 turns before being interrupted by a video sequence (i.e., unrelated game instruction or intervention, see Stimuli and Fig. 2) and 6 comprehension questions. These questions (e.g., Q: “*Did* [Tony or Lisa] *play the same game that we just played?*”; one treated boy and two girls answered with “no” but accurately answered all remaining questions) verified whether a child understood the clip and was able to connect the information to the current experimental situation. The series of 6 questions was directly followed by 30 additional turns of the gambling task before participants collected their final payoff. The average duration of the experiment, measured from the moment the child enters the room to the collection of stickers, was approximately 23 minutes. A hypothesized improvement of estimates and performance of the intervention group within the last half of the game would suggest that the intervention was effective in reducing overconfidence.

### Measure

To analyze this aspect, a main dependent variable was constructed by computing the difference between payoffs from 10 cards in block *t* to those in block *t-1* and comparing it to the associated estimates given after block *t-1*. This so-constructed calibration variable is binary with a value of 1 representing overconfidence. For instance, if a child earned fewer stickers on block 2 than on block 1, but predicted they would earn more stickers on the second block, the overconfidence score would be 1. In other words, overconfidence materialized whenever a child assumed to win more stickers but the difference in payoffs was negative, i.e., the child lost stickers. Similarly, overconfidence is present whenever a participant stated to win about the same amount of incentives but lost more than one sticker instead. Responses of “I don’t know” were treated as a sign of calibration as the child acknowledged the high degree of uncertainty for the upcoming turns, i.e., these responses were given an overconfidence score of 0. It may be noted that this is a conservative way to measure excessive confidence, but one that is more robust and appropriate for the underlying population. The coding resulted in four overconfidence data points per participants. Binary indicators were used in similar studies with young decision-makers^15^. The robustness of this measure was tested with a miscalibration variable (four data points per child), ranging from −1 (underconfidence) to 1 (overconfidence). Underconfidence materialized whenever a child stated to win less stickers but actually won more. Similarly, participants were considered to be underconfident if they expressed to win about the same number of stickers but won more than one sticker instead. Miscalibration and overconfidence are highly correlated (*r* = 0.89).

A secondary measure was constructed by assessing the choices of selected decks (i.e., number of chosen safe cards divided by 10 [number of turns per block]; see Fig. 1). The coding follows a relative frequency distribution, ranging from 0 to 1, with one indicating that only safe cards were selected within a 10-turn block. The coding led to six data points for relative safe card selection per participant. Lastly, a continues indicator for the sum of stickers after each block was constructed. Payoffs were calculated based on the selected cards and the predefined order^19^. The coding led to six payoff data points per child.

All intentionally collected variables were used for the analysis or the construction of the aforementioned exhaustive set of measures. No observations from children who assented and played the game were excluded. The results of all tested models are stated below.

## Results

The following analyses are Bayesian (generalized) random slope linear mixed models under default objective priors that were fitted in Stata 16^25–27^. These multilevel estimations represent a more robust alternative to common statistical tools for experimental data, such as t-tests or analyses of variance^25,28^. Arguably, the most notable advantage of this approach for child development research is that the results are not driven by sample sizes and even a small number of observations can lead to valid findings. That being said, the quality and availability of prior information are essential when examining small samples and a check of sensitivity towards the chosen priors is indispensable^29^. The presented results do not rely on such information and objective priors, assuming a null effect, are consistently used throughout the paper. All presented graphs were fitted in the R package jags^30^ and rely on Bayesian estimations using Gibbs sampling, following Kruschke^31^ and Bååth^32^. A complete set of convergence criteria can be found as Supplementary Material, along with all test items, and the results of a robustness check. Despite the hierarchical structure and complexity, all models show excellent convergence criteria with potential scale reduction factors that never exceeded 1.009, a desirable degree of state changes as the Markov chain Monte Carlo (MCMC) algorithm walks, and good mixing. Hence, all results are deemed reliable and reported below.

**Figure 3:**
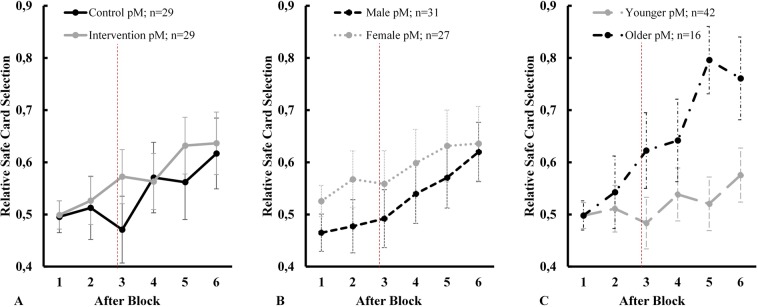
Posterior means (pM) for relative safe card selection by experimental arm (**A**), gender (**B**), and age (**C**) with vertical lines as posterior standard deviation (pSD). The vertical dashed red line represents the intervention that took place after block 3. All panels illustrate an increasing trend of relative safe card selection over time. Older children experienced the most notable rise, peaking at around 80% safe card selection on block 5.

The first step is to replicate findings from previous studies using the CGT. The results of a Bayesian random slope linear mixed model^27^ (as for all subsequent linear estimations, two MCMC chains computed in parallel, each with 10,000 iterations for burn-in, a thinning interval of five, 100,000 iterations to observe, random seed 12345, and Stata’s default objective priors with means of 0 and variances of 10,000 to reflect the high degree of uncertainty surrounding the selected null effect priors) to estimate the relative safe card selection over time, strongly suggest that girls have a higher likelihood to choose relatively more safe (compared to risky) cards, after controlling for intervention, age, and time (measured in blocks) fixed effects (posterior mean (pM) 11.7%, posterior standard deviation (pSD) 5.1%, region of practical equality (ROPE)^31^ 0 ± 0.01 = 1.12%; Column (1) in Table 1). In other words, an average of 98.88% of the mass of the posterior distribution of female fixed effects is larger 0.01. This means that 98.88% of the credible values indicate that, holding everything else constant, girls picked relatively more safe cards than boys. In comparison, 1.12% of the values are weak evidence that there is no (i.e., a mean effect size of 0) or, at least, no meaningful effect (i.e., ROPE of mean ± 0.01). Furthermore, participants seem to increase the relative number of safe cards picked over time (pM 2.5%, pSD 1.3%, ROPE 0 ± 0.01 = 11.14%). This finding is visually supported by Fig. 3 and in line with previous CGT studies that analyzed similar samples^19,22^. However, since 11.14% of the posterior mass lie between 0 and 0.01, the effect may not be particularly meaningful.

**Table 1:** Bayesian (Generalized) Linear Mixed Models with Random Intercepts and Slopes.

	(1) Relative Safe Cards pM (pSD) *[95% HPD]*	(2) Payoffs pM (pSD) *[95% HPD]*	(3) Overconfidence pM (pSD) *[95% HPD]*
**Fixed Effects**			
Intervention	**0.053** (0.059) *[−0.063; 0.168]*	**1.969** (1.078) *[−0.128; 4.071]*	**−0.111** (0.342) *[−0.791; 0.552]*
Age	**0.037** (0.036) *[−0.034; 0.108]*	**0.145** (0.692) *[−1.212; 1.506]*	**−0.221** (0.236) *[−0.693; 0.233]*
Female	**0.117** (0.051) *[0.017; 0.219]*	**2.867** (0.991) *[0.916; 4.817]*	**−0.303** (0.360) *[−1.020; 0.402]*
Time	**0.025** (0.013) *[0.000; 0.051]*	**0.080** (0.041) *[−0.001; 0.162]*	**−0.585** (0.135) *[−0.866; −0.332]*
**Interaction Terms**			
Intervention, Age, Blocks	**0.001** (0.004) *[−0.006; 0.008]*	**−0.001** (0.012) *[−0.024; 0.022]*	
Female, 2^nd^ Estimate			**−0.758** (0.369) *[−1.506; −0.051]*
Female, 4^th^ Estimate			**1.042** (0.479) *[0.122; 1.991]*
Constant	**0.196** (0.181) *[−0.161; 0.551]*	**−1.801** (3.469) *[−8.652; 5.007]*	**2.412** (1.211) *[0.107; 4.872]*
**Random Effects**			
Intercept	**0.014** (0.006) *[0.005; 0.029]*	**0.892** (1.137) *[0.110; 4.224]*	**0.358** (0.385) *[0.009; 1.358]*
Slope	**0.003** (0.001) *[0.002; 0.004]*	**0.042** (0.008) *[0.028; 0.061]*	**0.151** (0.099) *[0.018; 0.391]*
Variance	**0.040** (0.004) *[0.033; 0.047]*	**17.413** (1.487) *[14.719; 20.573]*	
N	58	58	58
Obs. per Participant	6	6	4

Note: Posterior mean (pM) in bold, posterior standard deviation (pSD) in parenthesis, 95% highest posterior density (HPD) interval in brackets and italics. Columns (1) and (2) are random slope Bayesian linear mixed models^27^. Column (3) is a random slope Bayesian generalized linear mixed model^26^.

There is no notable difference between the control and intervention group. Similarly, if participants aged 5 years 0 months or older (n = 16; 43.75% female) are compared to their younger counterparts (n = 42; 47.62% female), no age effect can be isolated after controlling for time, gender, and group allocation. Figure 3C indicates a more pronounced increase in relative safe card selection among older children after the third block, i.e., after the intervention group saw the video clip. An interaction between intervention, age, and time (measured in blocks) yields a null effect (ROPE 0 ± 0.01 = 99.21%).

**Figure 4:**
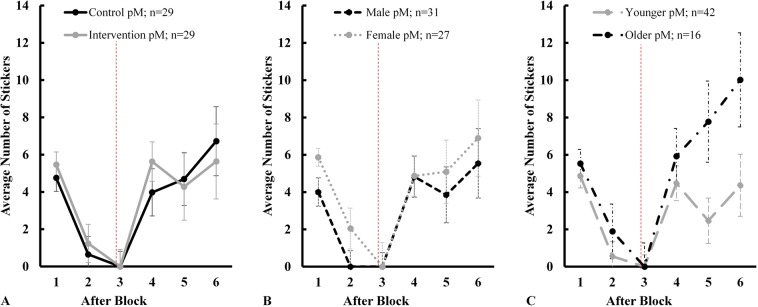
Posterior mean (pM) payoffs by experimental arm (**A**), gender (**B**), and age (**C**) with vertical lines as posterior standard deviation (pSD). The vertical dashed red line represents the intervention that took place after block 3. All panels illustrate a v-shaped curve for the average payoffs during the game. The recovery after block 3 seems sufficiently independent of the intervention.

More important for participants, however, is the number of stickers they won. Payoffs were also explicitly targeted by the video intervention. That is, both narrator and protagonist mentioned the loss of stickers; more or less advantageous deck selection was not mentioned during the short clip. Each participant started off with four stickers after the initial six practice trials. On average, every participant gained 0.3 stickers per turn and left the game with an average of 6.67 stickers, ranging from zero to 33. The lower bound was set to zero, i.e., participants could not have less than no stickers at any point during the game. Figure 4A illustrates the changes in payoffs per experimental arm after each block. For treated participants, there is a notable increase in gained stickers between the third and fourth block, i.e., pre and post video sequence. After accounting for age, gender, and time trends, these gains do not materialize in a strictly positive highest posterior density (HPD) interval (Column (2) in Table 1; pM = 1.97 stickers, pSD = 0.15). That said, it is worth noting that, unlike a frequentist confidence interval, the HPD is not an equal-tail interval. A ROPE ± 0.5 indicates that, on average, 92.4% of the posterior mass is larger than a 0.5 sticker increase. Despite this, any effect related to the video sequence seems to quickly wear off as both groups finished the experiment with approximately the same number of stickers.

A reoccurring finding is the presence of a gender effect. That is, the distribution of payoffs shows substantial variation by gender (Fig. 4B). Holding everything else constant, girls seem to outperform boys by an average of 2.87 stickers (pSD = 0.99, ROPE ± 0.5 = 0.81%). Age, time, and an interaction between intervention, age, and time (see Supplementary Fig. 1 for an illustration) do not seem to have any meaningful effect on the number of stickers won. The reason for the absence of a time trend can be found in the v-shaped curve of payoffs over time. Many children started and finished the experiment with roughly the same number of stickers. Consequently, there is no positive or negative trend line but rather a pattern of rapid losses and subsequent recoveries.

**Figure 5:**
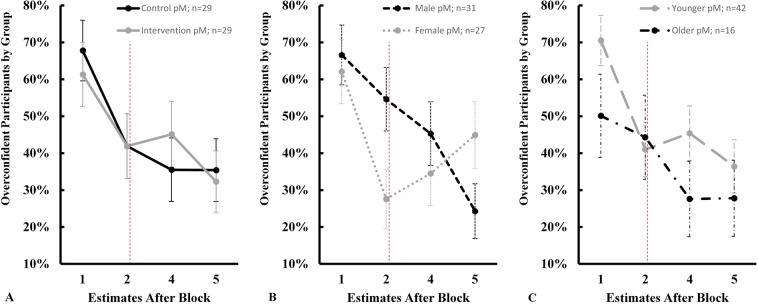
Posterior average proportion (pM) of overconfident participants by experimental arm (**A**), gender (**B**), and age (**C**) with vertical lines as pSD. The vertical dashed red line represents the intervention that took place between the second and third estimate (i.e., after block 3). While panels A and B illustrate a downward sloping trend, panel B suggests that female participants reversed this trend after the second estimate. Boys, on the other hand, monotonically decreased their overconfidence levels over time, indicating the presence of an early-age gender effect.

Lastly, the binary overconfidence variable is analyzed by using a Bayesian generalized linear mixed model with a random slope as well as a probit estimator, two MCMC chains computed in parallel, 10,000 iterations for burn-in, 400,000 iterations per chain to monitor, thinning of 20, and a random seed of 1234^26^. Default objective priors, that are by nature more informative than the previously used priors, given the underlying Bernoulli distribution of the dependent overconfidence variable, are applied^32^. Figure 5 provides a visual intuition for the overall development of overconfidence over time with payoff estimates after the first, second, fourth, and fifth block, each for the subsequent 10 turns.

Column (3) in Table 1 suggests that there is no traceable disparity between the intervention and control groups after accounting for fixed age, gender, and time effects (pM =−11.1%, pSD = 34.2%). Similarly, there seems to be little to no overall difference in overconfidence levels by age and gender over the course of the experiment. However, children appear to be less excessively confidence with each subsequent estimate, as proposed by the substantial time fixed effect (pM = −58.5%, pSD = 13.5%, ROPE ± 0.1 = 0.0025%). In other words, almost the entire (99.9975%) posterior mass indicate a time-dependent reduction of overconfidence, irrespective of group allocation, age, and gender. A visual explanation for a lack of an overall gender effect can be found in Fig. 5B. After playing the first 10 turns, girls and boys showed no initial sign of discrepancy in terms of their overconfidence levels. This seemingly supports previous studies that did not find any gender effect^3–6,8,9^. However, after 10 more cards, girls substantially decreased their excessive confidence levels only to then experience a sharp increase in overconfidence for the following 10 turns. Boys, on the other hand, gradually decreased their average excessive confidence over the course of the experiment. An interaction term between being female and the second (pM = −75.8%, pSD = 36.9%, ROPE ± 0.1 = 2.52%) and fourth (pM = 104.2%, pSD = 47.9%, ROPE ± 0.1 = 1.45%) estimates supports the finding that there is a substantial time-dependent gender effect. A robustness check, using a Bayesian linear mixed model with miscalibration (instead of binary overconfidence) as dependent variable, supports all reported findings regarding the impact of time, gender, and intervention on misplaced confidence (Supplementary Table 1). Therefore, the results seem sufficiently robust against parametric and operational changes.

## Discussion

The current study examines the presence and impact of overconfidence among 4- to 6-year-olds in a double-blind randomized controlled experimental setting with repeated trials, feedback, performance-based incentives, as well as with an informative vs uninformative video sequence. The outcome suggests that the vast majority of 4-year-olds (~70%) and half of all 5- and 6-year-olds are overconfident in their expectations after playing 10 turns (in addition to the 6 practice trials). This supports previous findings using metamemory tasks^3^. The data also seem to back claims that there is no underconfidence with practice (UWP) even after playing 60 trials as there was no notable increase in underconfidence over time and the absolute number of underconfident participants never exceeded 6 children within an experimental arm at any point in time^5,9^. In addition, the study suggests that children increase the number of safe cards they pick over time. This underlines prior findings regarding the development of hot executive functions using the CGT^19^.

However, unlike other papers using the same card game, the presented results do not assume that decision-makers optimize the selection of safe vs risky cards. Instead, the explicit focus is on payoffs and children’s expectations regarding the development of their payoff balance. While relative safe card selection and payoffs are interconnected, they represent different theoretical concepts. From an economic standpoint, children try to maximize their utility by winning stickers. One approach to achieve this would be to constantly pick relatively more safe cards. However, the order of the cards as well as the total number of turns are unknown to participants. If two children play 60% safe cards in any given block, their payoffs might still differ because the order in which the cards are played matters. This is the central element of uncertainty that differentiates the current experiment from previous metamemory studies. The diverging character of relative safe card selection and payoffs is visually supported by a comparison between Figs. 3 and 4. On average, children steadily increased the ratio of safe to risky cards over time. However, payoffs did not develop in the same fashion. For instance, older participants further increased their payoffs during the last 10 turns even though they decreased relative safe card selection. Future research may investigate the impact of gambling on this diverging pattern.

Furthermore, the current study provides insight into the impact of external feedback through an informative video sequence that essentially provides treated participants with an outlook on what might happen if they consistently choose the risky option. Contrary to a hypothesized decrease in excessive confidence, there was no meaningful intergroup difference between pre- and post-intervention. If anything, the insignificant increase experienced by the intervention group after seeing the video may be attributed to the more pronounced volatility among 4-year-olds whose confidence seems to be induced by the notable increase in payoffs during the fourth block.

Additionally, the findings shed light on a previously underreported aspect of an early-age gender effect in this process. Girls appear to substantially outperform boys in terms of relative safe card selection and payoffs. However, these effects do not materialize in any notable overall difference in overconfidence between girls and boys. Since overconfidence is a function of confidence and performance, the findings suggest that female participants increase their expectations towards future payoffs as they win more stickers. In other words, after increasing their payoffs on any given previous block, girls expected to win even more in the subsequent 10 turns. Otherwise, girls would gradually calibrate and eventually become underconfident with practice. Yet, this is not reflected by the data. On the contrary, Fig. 5 illustrates that girls adjust much more rapidly based on their performance during the first 20 cards of the game. As a consequence, they experienced the most visual drop in overconfidence among any of the comparison groups. However, girls’ self-assessment appears to be closely linked to their payoffs. While boys gradually decreased their overconfidence despite higher overall gains in the last 30 turns, the sampled girls reacted more volatilely and on par with their payoff balance. These deviating patterns, reflected by the substantial time x gender interaction in Table 1 Column (3), led to relatively more overconfident girls than boys by the end of the experiment; a finding that contradicts previous reports regarding more calibrated girls in metamemory tasks^7^. It may also be noted that whenever a substantial gender effect emerged (i.e., after the second and fifth block), payoffs between male and female participants were not vastly different (Fig. 4B).

The impact of age might be less pronounced than in previous studies using metamemory tasks^3,6,8,9^. Younger and older children did not differ in terms of their relative safe card selection, payoffs, or overconfidence levels after controlling for intervention, gender, and time fixed effects. The advantage of more developed cognitive functions among 5- and 6-year-olds might have only played out in the final two blocks when they vastly outperformed their younger peers (Figs. 3C and 4C). However, this pattern did not translate into a more calibrated self-assessment. In fact, the only notable difference between the two age groups emerged after the first block. It may well be that task novelty is the primary reason for this initial discrepancy, as 4-year-olds quickly adjusted their prediction for the following block.

A limitation of the present study is the lack of diversity in the underlying sample that mainly consists of children from families of high socioeconomic status. A more regionally, ethnically, and socially diverse set of participants would have been highly desirable. In addition, future research may want to consider deviating from the previously imposed 10-turn-per-block structure in order to generate more data points for the confidence estimates. While the applied block structure did allow to replicate findings from previous CGT studies, the novelty and ultimate purpose of this study was to adjust the procedure of the CGT in order to assess overconfidence among young decision-makers and more observations in this regard would have been preferable. Lastly, a lack of engagement with the illustrated video sequence might have been responsible for the absence of an intervention effect. In the current study, experimenters did not engage with participants during the intervention in order to minimize experimental noise. However, future research may want to consider enhancing the stimuli with a guided reflection task.

Nevertheless, the implications of this study are manifold. First, overconfidence is persistent and widespread during early childhood. Approximately one out of four 5- to 6-year-old and every third 4-year-old are overconfident even after a vast number of repetitions and feedback. Second, displaying a suboptimal strategy in the form of a video intervention does not seem to have any lasting effect. Third, boys seem to adjust their inflated predictions more gradually and independent of their payoffs compared to girls who, contrary to previous findings, exhibit more volatility in their self-assessments and a higher propensity for overconfidence in case of increasing gains^2,7^. This aspect should be considered in future child development studies on overconfidence. The presented findings advance the fields of developmental and behavioral sciences by shedding light on how children (over)estimate their own performance over a large number of repetitions and in a situation of risk and uncertainty.

## Supplementary information

Supplementary information

## Data Availability

The data and survey that support the findings of this study are available at UK Data Service ReShare (ID 854238).
