# Supplementary material for: Overconfidence Among Young Decision-Makers: Assessing the Effectiveness of a Video Intervention and the Role of Gender, Age, Feedback, and Repetition
Source: Sci Rep. 2020 Mar 4;10:3984. doi: 10.1038/s41598-020-61078-z (PMC7055226; doi:10.1038/s41598-020-61078-z)
Supplement: Supplementary file 1 — Supplementary information [file 41598_2020_61078_MOESM1_ESM.pdf]

Online Supplementary Material

Overconfidence Among Young Decision-Makers: Assessing the Effectiveness of a Video Intervention and the Role of Gender, Age, Feedback, and Repetition

Dominik M. Piehlmaier, University of Wisconsin-Madison

Current Affiliation: University of Sussex Business School

This is the online supplement to the manuscript "Overconfidence Among Young Decision-Makers: Assessing the Effectiveness of a Video Intervention and the Role of Gender, Age, Feedback, and Repetition". The web appendix is structed as follows. First, a complete copy of the experimenter input survey is provided. This includes links to the video sequences, the randomization, the survey flow, and an exhaustive set of observational items that were used for the study. Second, a copy of the experimenter script is provided. Third, a copy of all four decks is presented. Fourth, a supplementary figure and table are included to check the robustness of the results in the main article. Finally, an array of supplementary figures for the utilized models are illustrated in order to provide graphic evidence for convergence and mixing of the MCMC algorithm.

Survey Input and Flow

Block: Validation (5 Questions)

Block Randomizer: 1 - Evenly Present Elements

Standard: Clinical (29 Questions)
Standard: Control (29 Questions)

Page Break

Start of Block: Validation

Q70 Forgot your script? Follow this link [deactivated; find script below] to download it.

Q1 RA Number

____

Q2 Parent's Email (see Consent Form)

____

Q3 Child's gender

- ○ Male (1)
- ○ Female (2)

Q65 Did the child give oral assent?

- ○ Yes (1)
- ○ No (2)

End of Block: Validation

Start of Block: Clinical

Q4 CLIN

Q5 Deck Arrangement: Place sticker sheet between the two decks
"Dots" **to your left**

Q6 Deck Arrangement: Place sticker sheet between the two decks
"Stripes" **to your left**

Q66 Deck Arrangement: Place sticker sheet between the two decks
"Triangles" **to your left**

Q67 Deck Arrangement: Place sticker sheet between the two decks
"Waves" **to your left**

Q7 Play ten turns. Mark each time which deck has been selected.
Ask estimation question once ten cards have been displayed.

	Safe Deck (1 Smiley face win) (1)	Risky Deck (2 Smiley faces win) (2)
1.Turn (1)	○	○
2.Turn (2)	○	○
3.Turn (3)	○	○
4.Turn (4)	○	○
5.Turn (5)	○	○
6.Turn (6)	○	○
7.Turn (7)	○	○
8.Turn (8)	○	○
9.Turn (9)	○	○
10.Turn (10)	○	○

Q8 Estimation Question: "**Now, how much do you think you will win with the next 10 cards?**"

- More (1)
- About the same (2)
- Less (3)
- Don't know (4)

Q9 Play ten turns. Mark each time which deck has been selected.
Ask estimation question once ten cards have been displayed.

	Safe Deck (1)	Risky Deck (2)
1.Turn (1)	○	○
2.Turn (2)	○	○
3.Turn (3)	○	○
4.Turn (4)	○	○
5.Turn (5)	○	○
6.Turn (6)	○	○
7.Turn (7)	○	○
8.Turn (8)	○	○
9.Turn (9)	○	○
10.Turn (10)	○	○

Q10 Estimation Question: "**Now, how much do you think you will win with the next 10 cards?"**

- More (1)
- About the same (2)
- Less (3)
- Don't know (4)

Q11 Play ten turns. Mark each time which deck has been selected.
Ask estimation question once ten cards have been displayed.

	Safe Deck (1)	Risky Deck (2)
1.Turn (1)	○	○
2.Turn (2)	○	○
3.Turn (3)	○	○
4.Turn (4)	○	○
5.Turn (5)	○	○
6.Turn (6)	○	○
7.Turn (7)	○	○
8.Turn (8)	○	○
9.Turn (9)	○	○
10.Turn (10)	○	○

Display This Question:

If gender = Female

Q71 Please show the **entire video in full screen** by following one of the links below:

If you play with **stripes and dots**: https://youtu.be/MtH4nCBemmw

If you play with **triangles and waves**: https://youtu.be/m_0BzYY03uo

Display This Question:

If gender = Male

Q16 Please show the **entire video in full screen** by following one of the links below:

If you play with **stripes and dots**: https://youtu.be/TZw6HILJKFI

If you play with **triangles and waves**: https://youtu.be/xqNYOGv_9ZM

Display This Question:

If gender = Male

Q17 Did Tony play the same game that we just played?

- Yes (1)
- No (2)
- Don't know (3)

Display This Question:

If gender = Male

Q18 Did he win more than you?

- ◯ Yes (1)
- ◯ No (2)
- ◯ Don't know (3)

Display This Question:

If gender = Male

Q19 Why do you think he did not win as much?

- ◯ Always played risky deck (1)
- ◯ Always played safe deck (2)
- ◯ Other (3) ____
- ◯ Don't know (4)

Display This Question:

If gender = Male

Q20 What would you have done differently?

- ◯ Play safe deck (1)
- ◯ Nothing (2)
- ◯ Other (3) ____
- ◯ Don't know (4)

Display This Question:

If gender = Male

Q21 If we played the card game again, would you play like Tony?

- Yes (1)
- No (2)
- Don't know (3)

Display This Question:

If gender = Male

Q22 Why/Why not?

- Lost more often (1)
- Better player (2)
- Other (3) ____
- Don't know (4)

Display This Question:

If gender = Female

Q23 Did Lisa play the same game that we just played?

- Yes (1)
- No (2)
- Don't know (3)

Display This Question:

If gender = Female

Q24 Did she win more than you?

◯ Yes (1)

◯ No (2)

◯ Don't know (3)

Display This Question:

If gender = Female

Q25 Why do you think she did not win as much?

◯ Always played risky deck (1)

◯ Always played safe deck (2)

◯ Other (3) ____

◯ Don't know (4)

Display This Question:

If gender = Female

Q26 What would you have done differently?

◯ Play safe deck (1)

◯ Nothing (2)

◯ Other (3) ____

◯ Don't know (4)

Display This Question:

If gender = Female

Q27 If we played the card game again, would you play like Lisa?

- ◯ Yes (1)
- ◯ No (2)
- ◯ Don't know (3)

Display This Question:

If gender = Female

Q28 Why/Why not?

- ◯ Lost more often (1)
- ◯ Better player (2)
- ◯ Other (3) ____
- ◯ Don't know (4)

Q29 Play ten turns. Mark each time which deck has been selected.
Ask estimation question once ten cards have been displayed.

	Safe Deck (1)	Risky Deck (2)
1.Turn (1)	○	○
2.Turn (2)	○	○
3.Turn (3)	○	○
4.Turn (4)	○	○
5.Turn (5)	○	○
6.Turn (6)	○	○
7.Turn (7)	○	○
8.Turn (8)	○	○
9.Turn (9)	○	○
10.Turn (10)	○	○

Q30 Estimation Question: "**Now, how much do you think you will win with the next 10 cards?"**

○ More (1)

○ About the same (2)

○ Less (3)

○ Don't know (4)

Q31 Play ten turns. Mark each time which deck has been selected.
Ask estimation question once ten cards have been displayed.

	Safe Deck (1)	Risky Deck (2)
1.Turn (1)	○	○
2.Turn (2)	○	○
3.Turn (3)	○	○
4.Turn (4)	○	○
5.Turn (5)	○	○
6.Turn (6)	○	○
7.Turn (7)	○	○
8.Turn (8)	○	○
9.Turn (9)	○	○
10.Turn (10)	○	○

Q32 Estimation Question: "**Now, how much do you think you will win with the next 10 cards?**"

- More (1)
- About the same (2)
- Less (3)
- Don't know (4)

Q33 Play ten turns. Mark each time which deck has been selected.
This is the final round; proceed with script.

	Safe Deck (1)	Risky Deck (2)
1.Turn (1)	○	○
2.Turn (2)	○	○
3.Turn (3)	○	○
4.Turn (4)	○	○
5.Turn (5)	○	○
6.Turn (6)	○	○
7.Turn (7)	○	○
8.Turn (8)	○	○
9.Turn (9)	○	○
10.Turn (10)	○	○

End of Block: Clinical

Start of Block: Control

Q35 CON

Q36 Deck Arrangement: Place sticker sheet between the two decks
"Dots" **to your left**

Q37 Deck Arrangement: Place sticker sheet between the two decks
"Stripes" **to your left**

Q68 Deck Arrangement: Place sticker sheet between the two decks
"Triangles" **to your left**

Q69 Deck Arrangement: Place sticker sheet between the two decks
"Waves" **to your left**

Q38 Play ten turns. Mark each time which deck has been selected.
Ask estimation question once ten cards have been displayed.

	Safe Deck (1)	Risky Deck (2)
1.Turn (1)	○	○
2.Turn (2)	○	○
3.Turn (3)	○	○
4.Turn (4)	○	○
5.Turn (5)	○	○
6.Turn (6)	○	○
7.Turn (7)	○	○
8.Turn (8)	○	○
9.Turn (9)	○	○
10.Turn (10)	○	○

Q39 Estimation Question: "**Now, how much do you think you will win with the next 10 cards?"**

○ More (1)

○ About the same (2)

○ Less (3)

○ Don't know (4)

Q40 Play ten turns. Mark each time which deck has been selected.
Ask estimation question once ten cards have been displayed.

	Safe Deck (1)	Risky Deck (2)
1.Turn (1)	○	○
2.Turn (2)	○	○
3.Turn (3)	○	○
4.Turn (4)	○	○
5.Turn (5)	○	○
6.Turn (6)	○	○
7.Turn (7)	○	○
8.Turn (8)	○	○
9.Turn (9)	○	○
10.Turn (10)	○	○

Q41 Estimation Question: "**Now, how much do you think you will win with the next 10 cards?"**

- ○ More (1)
- ○ About the same (2)
- ○ Less (3)
- ○ Don't know (4)

Q42 Play ten turns. Mark each time which deck has been selected.
Ask estimation question once ten cards have been displayed.

	Safe Deck (1)	Risky Deck (2)
1.Turn (1)	○	○
2.Turn (2)	○	○
3.Turn (3)	○	○
4.Turn (4)	○	○
5.Turn (5)	○	○
6.Turn (6)	○	○
7.Turn (7)	○	○
8.Turn (8)	○	○
9.Turn (9)	○	○
10.Turn (10)	○	○

Display This Question:

If gender = Male

Q46 Please show the **entire video in full screen** by following the link below:

https://youtu.be/br_1qDppiKM

Display This Question:

If gender = Female

Q47 Please show the **entire video in full screen** by following the link below:

https://youtu.be/1ibKvv7n8ns

Display This Question:

If gender = Male

Q48 Did he talk about the same game that we just played?

○ Yes (1)

○ No (2)

Display This Question:

If gender = Male

Q49 Do you think the game he talked about is fun?

○ Yes (1)

○ No (2)

Display This Question:

If gender = Male

Q50 Is it more or less difficult than our card game?

- More difficult (1)
- About the same (2)
- Less difficult (3)
- Don't know (4)

Display This Question:

If gender = Male

Q51 Have you played that game before?

- Yes (1)
- No (2)
- Don't know (3)

Display This Question:

If gender = Male

Q52 Do you think your friends would like to play this game with you?

- Yes (1)
- No (2)
- Don't know (3)

Display This Question:

If gender = Male

Q53 Why/Why not?

____

Display This Question:

If gender = Female

Q54 Did she talk about the same game that we just played?

- Yes (1)
- No (2)

Display This Question:

If gender = Female

Q55 Do you think the game she talked about is fun?

- Yes (1)
- No (2)

Display This Question:

If gender = Female

Q56 Is it more or less difficult than our card game?

- More difficult (1)
- About the same (2)
- Less difficult (3)

Display This Question:

If gender = Female

Q57 Have you played that game before?

- ○ Yes (1)
- ○ No (2)
- ○ Don't know (3)

Display This Question:

If gender = Female

Q58 Do you think your friends would like to play this game with you?

- ○ Yes (1)
- ○ No (2)
- ○ Don't know (3)

Display This Question:

If gender = Female

Q59 Why/Why not?

____

Q60 Play ten turns. Mark each time which deck has been selected.
Ask estimation question once ten cards have been displayed.

	Safe Deck (1)	Risky Deck (2)
1.Turn (1)	○	○
2.Turn (2)	○	○
3.Turn (3)	○	○
4.Turn (4)	○	○
5.Turn (5)	○	○
6.Turn (6)	○	○
7.Turn (7)	○	○
8.Turn (8)	○	○
9.Turn (9)	○	○
10.Turn (10)	○	○

Q61 Estimation Question: "**Now, how much do you think you will win with the next 10 cards?"**

○ More (1)

○ About the same (2)

○ Less (3)

○ Don't know (4)

Q62 Play ten turns. Mark each time which deck has been selected.
Ask estimation question once ten cards have been displayed.

	Safe Deck (1)	Risky Deck (2)
1.Turn (1)	○	○
2.Turn (2)	○	○
3.Turn (3)	○	○
4.Turn (4)	○	○
5.Turn (5)	○	○
6.Turn (6)	○	○
7.Turn (7)	○	○
8.Turn (8)	○	○
9.Turn (9)	○	○
10.Turn (10)	○	○

Q63 Estimation Question: "**Now, how much do you think you will win with the next 10 cards?"**

- More (1)
- About the same (2)
- Less (3)
- Don't know (4)

Q64 Play ten turns. Mark each time which deck has been selected.
This is the final round; proceed with script.

	Safe Deck (1)	Risky Deck (2)
1.Turn (1)	○	○
2.Turn (2)	○	○
3.Turn (3)	○	○
4.Turn (4)	○	○
5.Turn (5)	○	○
6.Turn (6)	○	○
7.Turn (7)	○	○
8.Turn (8)	○	○
9.Turn (9)	○	○
10.Turn (10)	○	○

End of Block: Control

Experimenter Script

Supplies: Cards (four decks with 70 cards each); set of stickers; one sticker sheet

Preparations: Make sure all cards are in order (see Kerr & Zelazo, 2004, p. 151 for the first 50 cards; start from the beginning for the remaining 20 cards). Open the Qualtrics survey using the link that was provided to you in an email and put in your two-digit RA number. Place the two decks according to the directions in the survey, the sticker sheet should be placed between the two decks. Follow the prompts from the survey; you will need the parent's consent form to proceed (make sure to put in the correct email address).

Directions: REMEMBER, you are the researcher; do not influence or change the child's behavior. Every experiment must be equal to the one before – even between different RAs.

"**Do you like stickers? Well, we are going to play a game where you get to win stickers. Do you want one?"** [Give one STICKER to the child so that the children know what they are playing for.]

"Okay, in this game we get to put the stickers that you win on this sheet. All stickers that are left on the sheet after the game are yours to keep." [point towards the sticker sheet]. **"I'll show you how the game works and how you can win some more**."

[A training session is then given to the child, where the child will be introduced to the game by demonstrating what happens when the first 3 cards from each deck are turned, i.e. 3 from one deck first and then 3 from the other deck:] e.g. 1st turn.: **"Okay, I'm going to show you how the game works. We have the stripe [triangle] cards and the dot [wave] cards. Let's see how the dots [waves] work first."** [Flip the card.] "**Look there's 1 happy face, that means that you**

win 1 sticker "[Place 1 sticker on top of the happy face counting out loud, and then place it on the sticker sheet.]

"Okay, now we have to open this up and see if there's any sad faces. "[The bottom part of the cards is covered with a yellow sheet of Post-It paper, so that the children do not get distracted with the losses right away. **Wins** are given out **first** and then the losses are taken.]

"Oh there's 1 sad face, which means that you lose 1 sticker. So, we have to give 1 sticker back." [Take 1 sticker from the sheet and place it on top of the sad face counting out loud and then place it back to the original set of stickers] "**We don't like the sad faces, do we? Because we lose stickers, but we like the happy faces, right? Because we get to win stickers!**

[After the training part]: **"Okay, now we are ready to start playing the game. You get to choose whichever card you want to play with every time. You can play from the dots or the stripes each turn. You get to choose one card every time and you can pick as many cards as you want until I say STOP and then the game will be over. So, remember you want to make sure that you win as many stickers as possible! Let's see if we can fill this whole sheet with stickers! Whatever you have on the sheet by the end of the game, you can keep and take home with you. Okay? Which card do you want to flip first?"**

[Mark every selection after the 6 training turns in the survey. Continue until 60 cards have been turned. After every 10 rounds, ask whether the children think that they will win (1) more, (2) about the same (~ +/- 1), or (3) less stickers in the following 10 turns; the answer "Don't know" can be selected but must not be given as an option to the child. Make a note in the survey]: **"Alright, let's see how much you won with the last 10 cards. WOW, great! Now, how much do you think you will win with the next 10 cards? More, about the same, or less stickers?"** [After the first 30 turns, proceed with the video. Let the child watch the entire video without

interruption. Ask the child the questions provided in the survey, once the clip is over.] **"That was interesting, wasn't it?"** [Ask the 6 comprehension questions and continue with the last 30 turns]

[Also, if the losses outnumber the number of stickers on the sheet, just take back everything that is on the sheet and explain why]: **"We don't even have that many to give back, so we'll have to give back everything we have."** [Leaving the sticker sheet empty.]

-At the end of the game, the child is rewarded with an extra sticker for having done **"such a good job."**

-Also, let the child deposit the wins and give back the losses for the first 6 card turns to get them involved, but after that, if the experimenter controls the handling of rewards and losses, the task will go **much** smoother and **faster**.

Cards

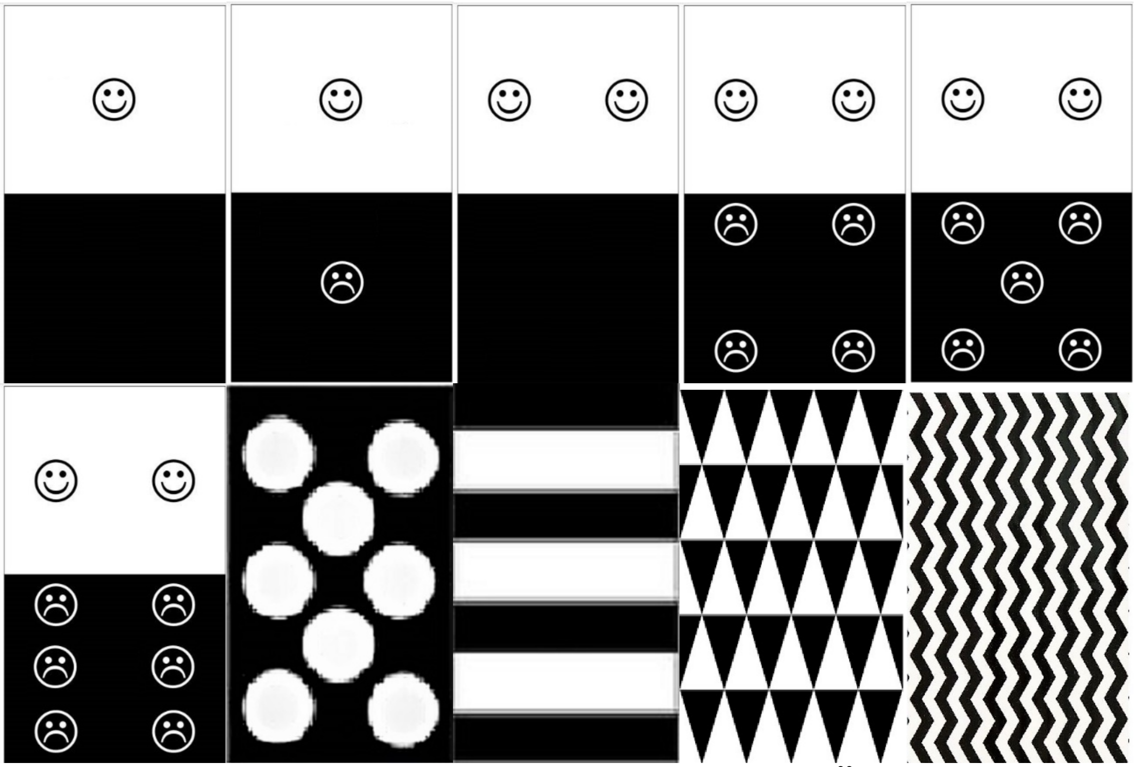

Scale: 1:3; Actual Dimensions: 15 x 9 cm. For Stripes and Dots see Faja et al. [22] (License Number 4744400415515)

Supplementary Figure

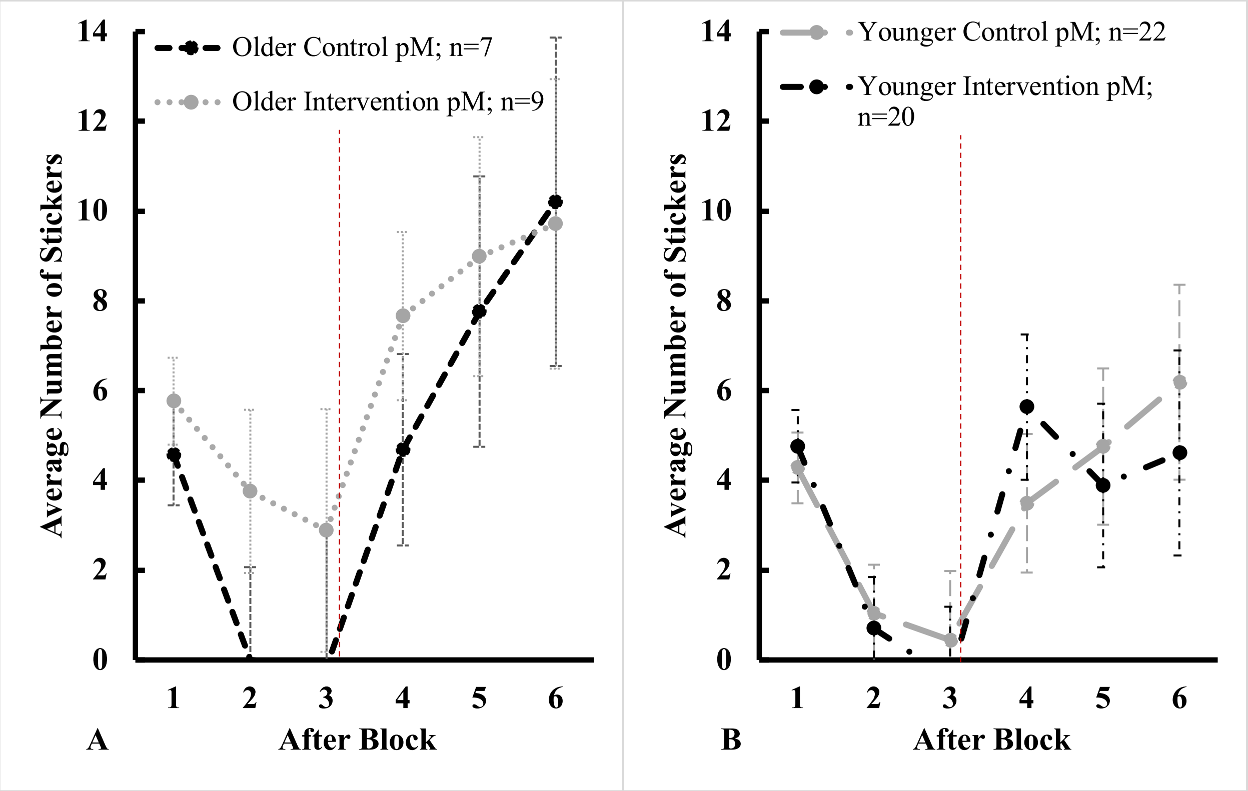

Supplementary Figure 1. Posterior mean (pM) payoffs by interaction between age and intervention with vertical lines as posterior standard deviation (pSD). The vertical dashed red line represents the intervention that took place after block 3.

Supplementary Table

Supplementary Table 1 Robustness Check Replacing Binary Overconfidence with Ordinal Miscalibration

	Miscalibration<br>**pM**<br>(pSD)<br>*[95% HPD]*
**Fixed Effects**	
Intervention	**0.019**<br>(0.121)<br>*[-0.220; 0.253]*
Age	**-0.014**<br>(0.083)<br>*[-0.177; 0.148]*
Female	**-0.152**<br>(0.156)<br>*[-0.457; 0.155]*
Time	**-0.181**<br>(0.051)<br>*[-0.280; -0.081]*
**Interaction Terms**	
Female, 2nd Estimate	**-0.338**<br>(0.166)<br>*[-0.667; -0.011]*
Female, 3rd Estimate	**-0.046**<br>(0.192)<br>*[-0.426; 0.330]*
Female, 4th Estimate	**0.246**<br>(0.228)<br>*[-0.201; 0.695]*
Constant	**0.912**<br>(0.423)<br>*[0.079; 1.748]*
**Random Effects**	
Intercept	**0.041**<br>(0.032)<br>*[0.005; 0.124]*
Slope	**0.014**<br>(0.006)<br>*[0.005; 0.028]*
Variance	**0.326**<br>(0.036)<br>*[0.262; 0.403]*
N	58
Obs. per Participant	4

Note: Posterior mean (pM) in bold, posterior standard deviation (pSD) in parenthesis, 95% highest posterior density (HPD) interval in brackets and italics. The robustness check is a random slope Bayesian linear mixed model.

Convergence and Robustness

The following exhaustive set of supplementary figures illustrates the reported distributions, trace-, and density plots for all presented model parameters in the main article and this online supplement, as well as their mixing. It may be noted that overconfidence measures were taken after the first, second, fourth, and fifth block. Each subheading represents the associated column in Table 1 and Supplementary Table 1

Relative Safe Card Selection

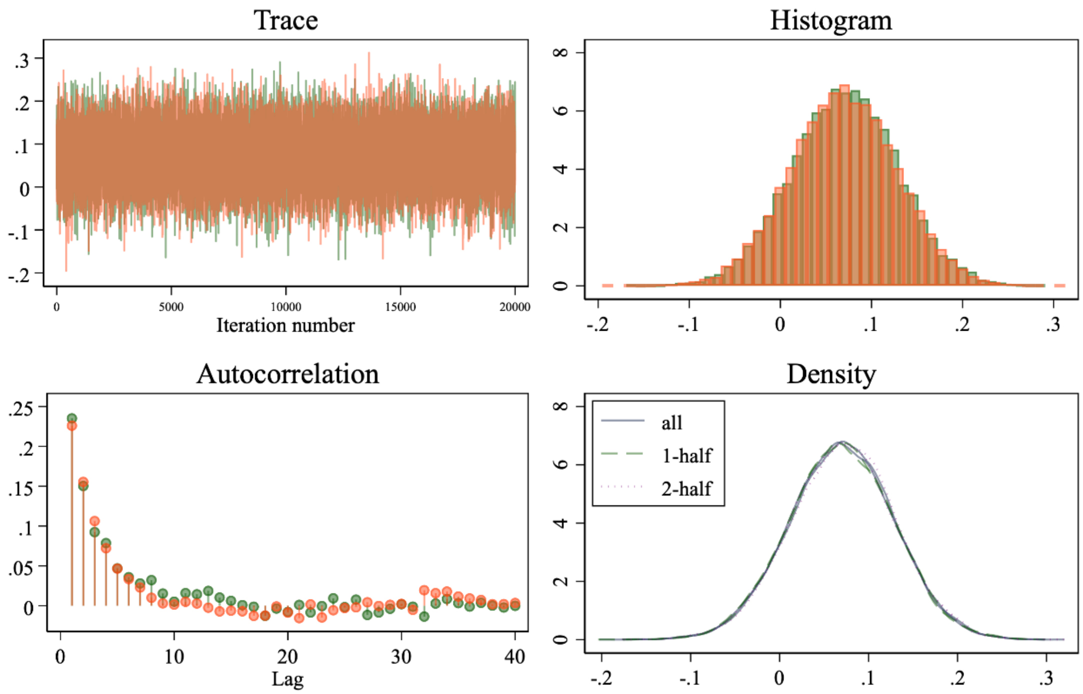

Supplementary Figure 2. Convergence Criteria for Relative Safe Card Selection; Intervention Parameter

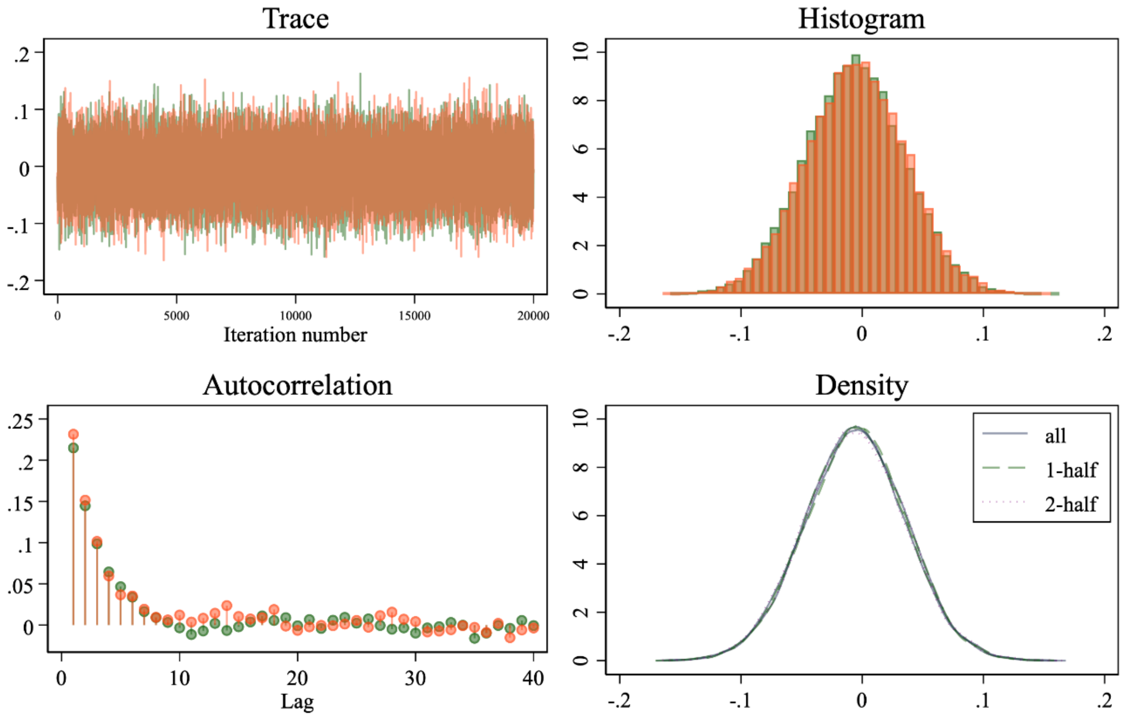

Supplementary Figure 3. Convergence Criteria for Relative Safe Card Selection; Age Parameter

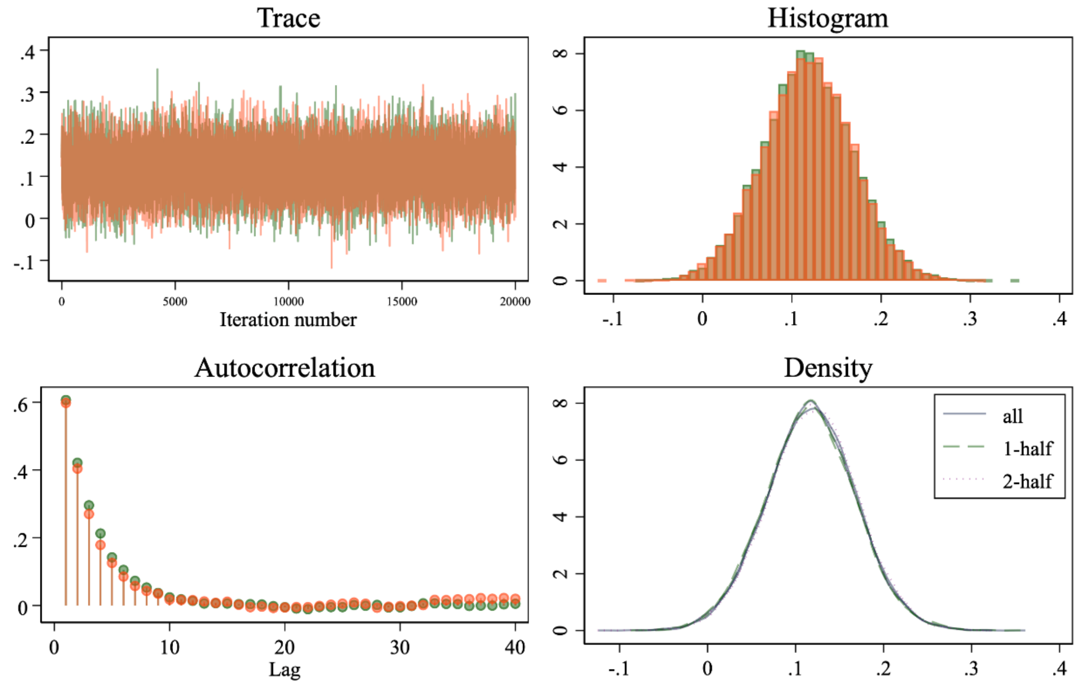

Supplementary Figure 4: Convergence Criteria for Relative Safe Card Selection; Gender Parameter

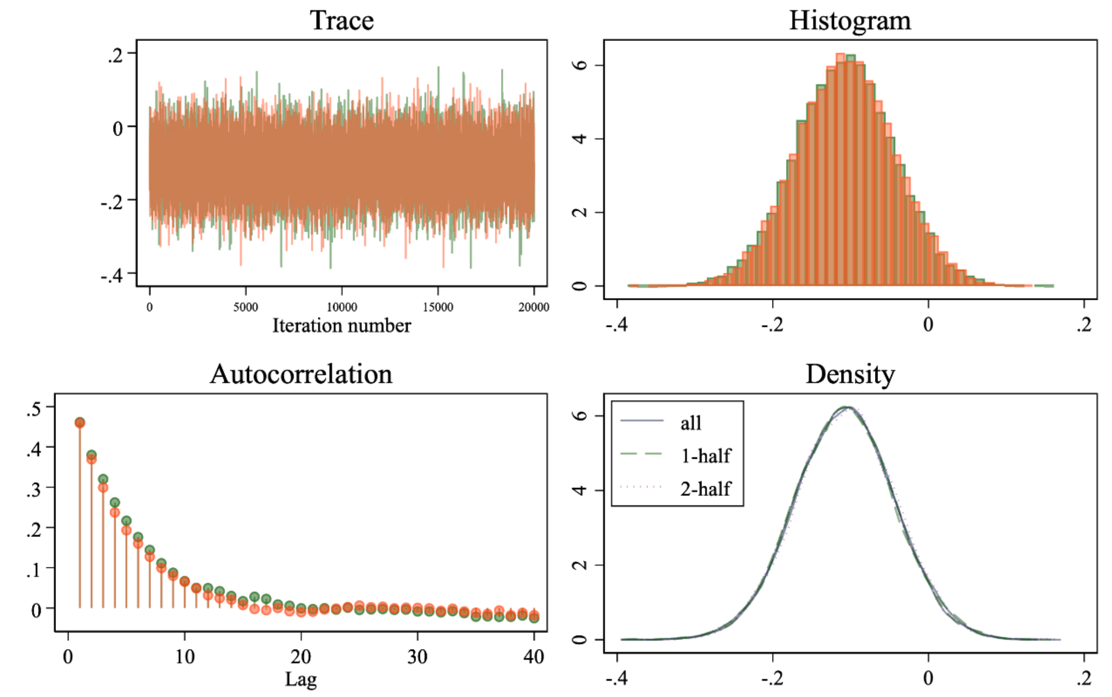

Supplementary Figure 5. Convergence Criteria for Relative Safe Card Selection; Time Parameter

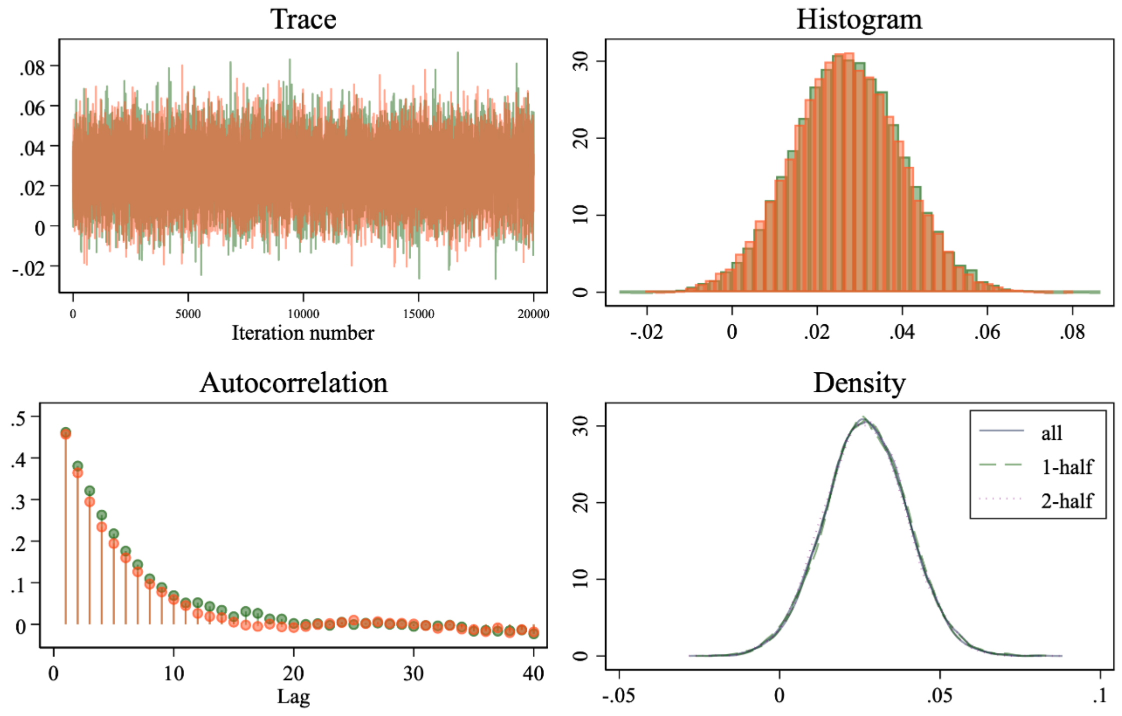

Supplementary Figure 6. Convergence Criteria for Relative Safe Card Selection; Interaction Parameter

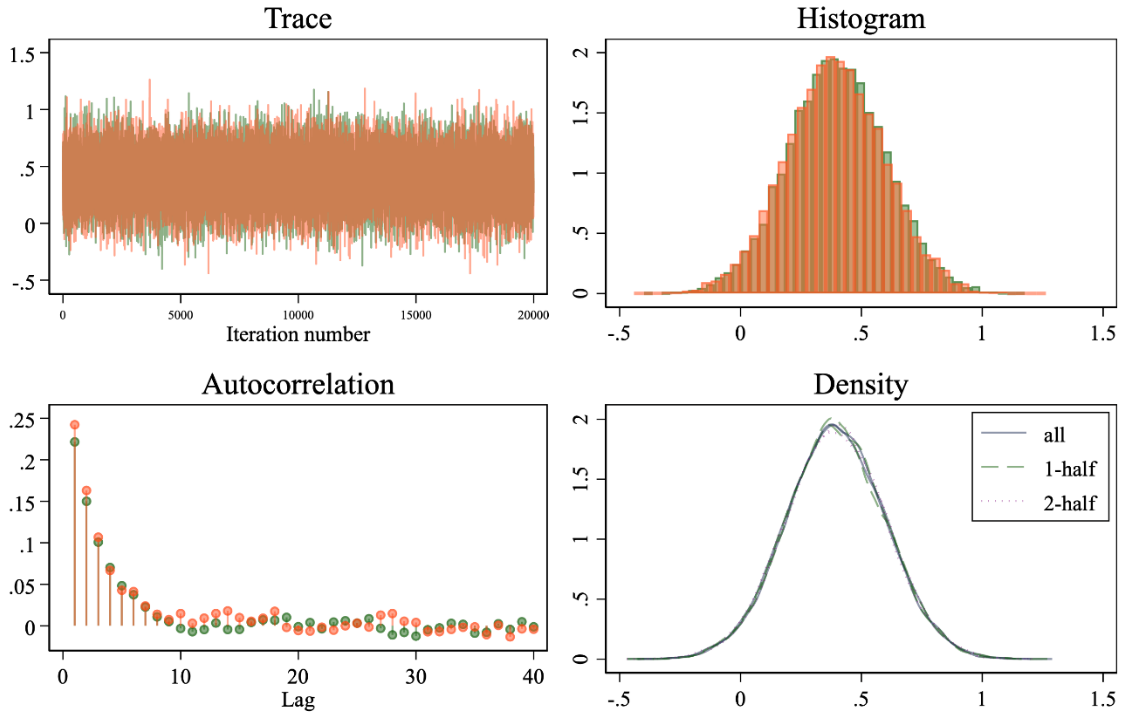

Supplementary Figure 7. Convergence Criteria for Relative Safe Card Selection; Constant Parameter

Payoffs

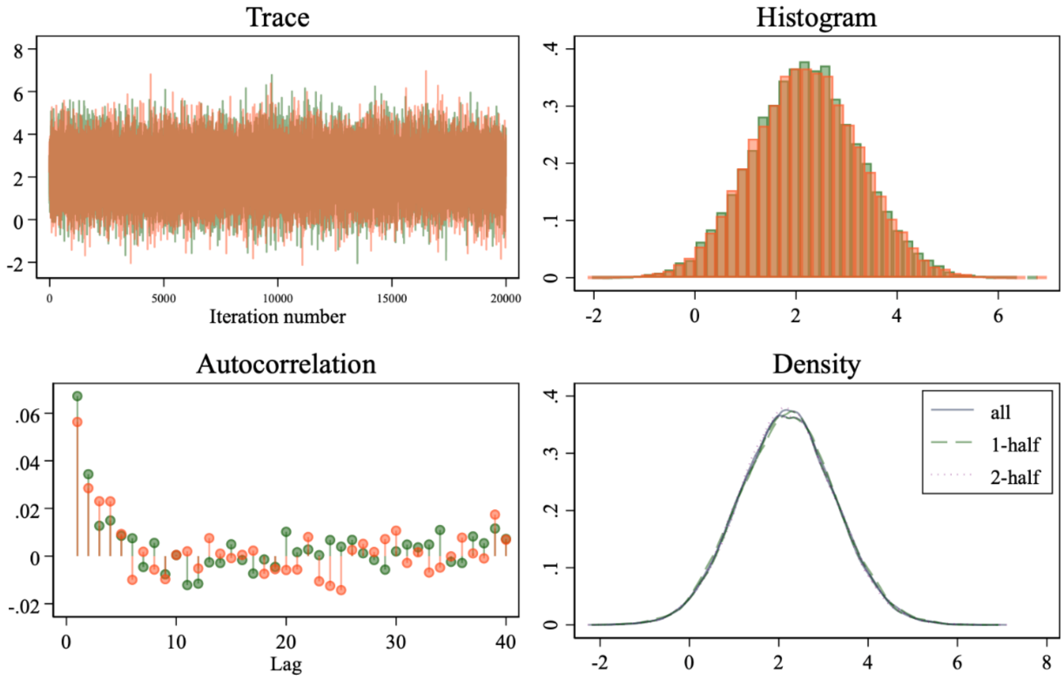

Supplementary Figure 8. Convergence Criteria for Payoffs; Intervention Parameter

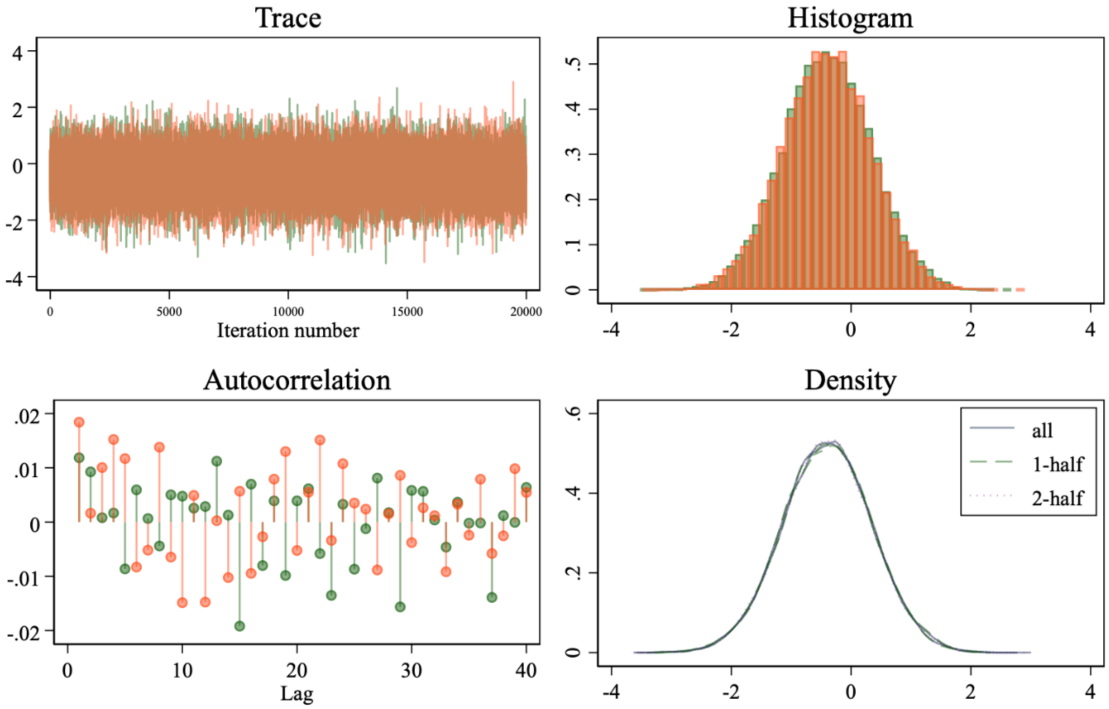

Supplementary Figure 9. Convergence Criteria for Payoffs; Age Parameter

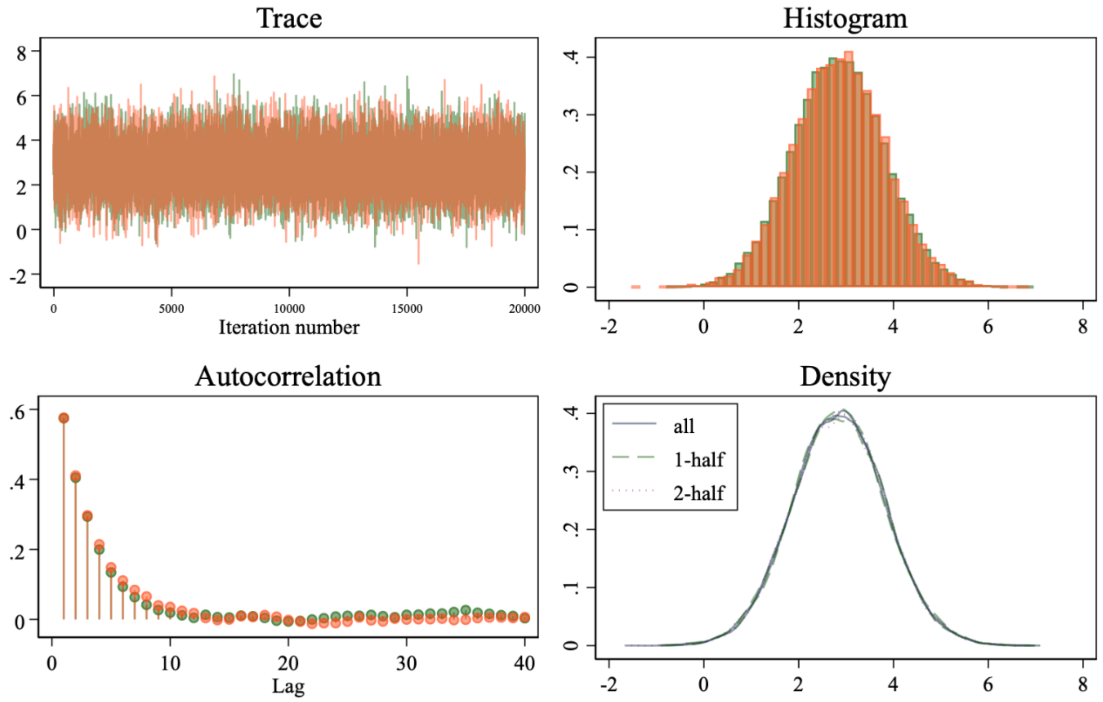

Supplementary Figure 10. Convergence Criteria for Payoffs; Gender Parameter

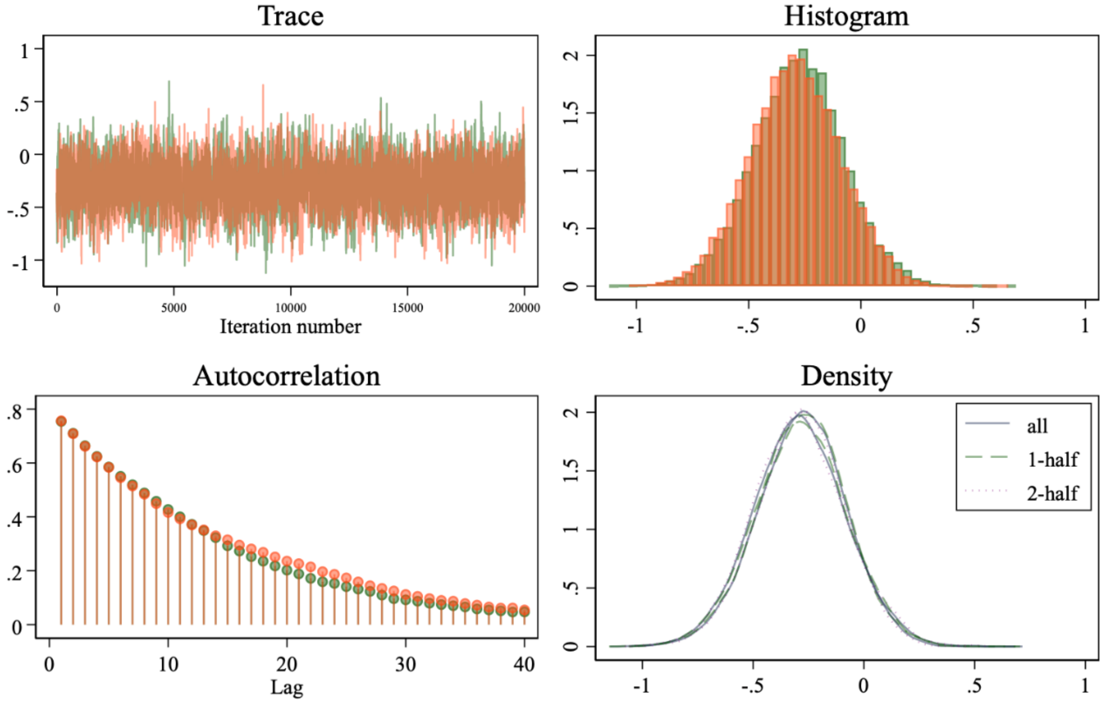

Supplementary Figure 11. Convergence Criteria for Payoffs; Time Parameter

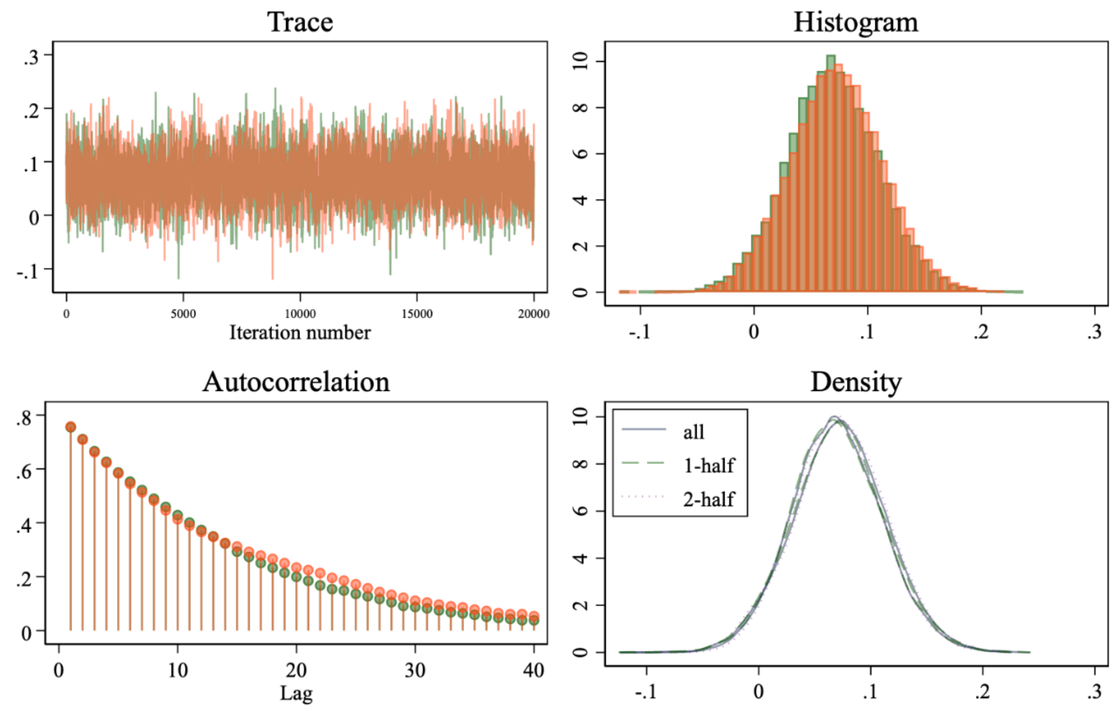

Supplementary Figure 12. Convergence Criteria for Payoffs; Interaction Parameter

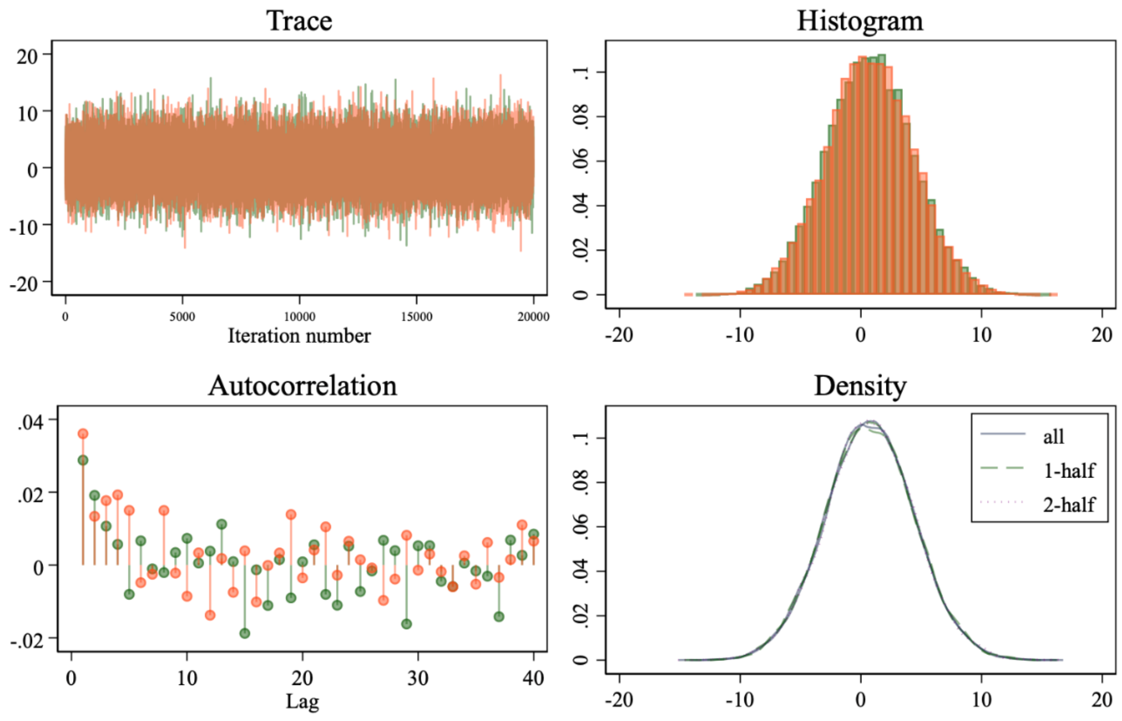

Supplementary Figure 13. Convergence Criteria for Payoffs; Constant Parameter

Overconfidence

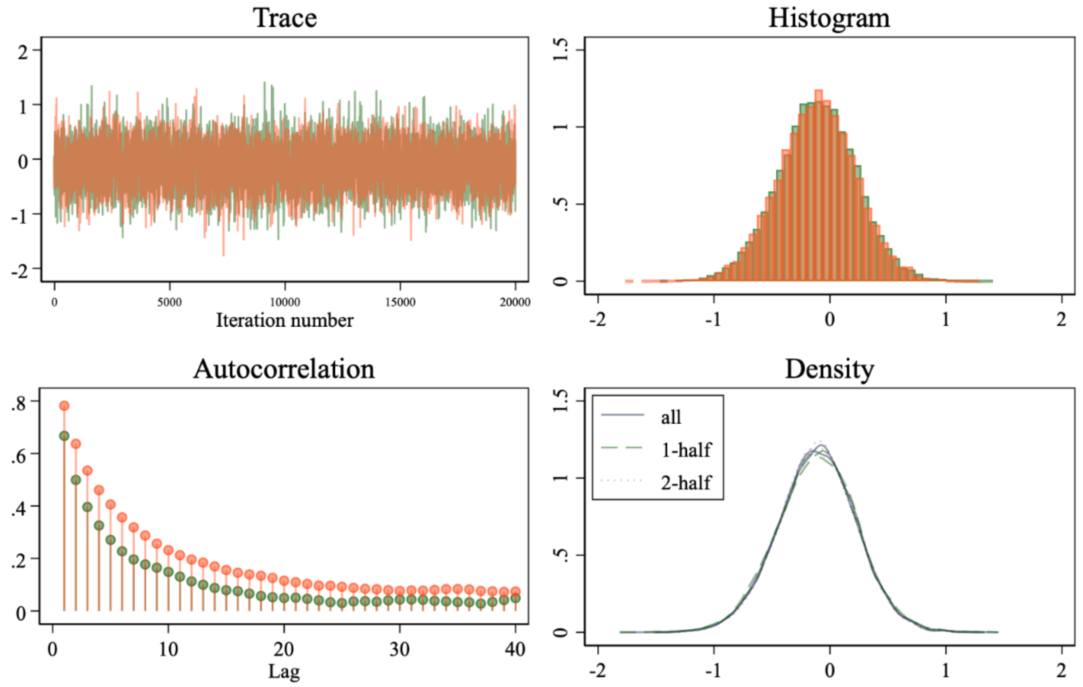

Supplementary Figure 14. Convergence Criteria for Overconfidence; Intervention Parameter

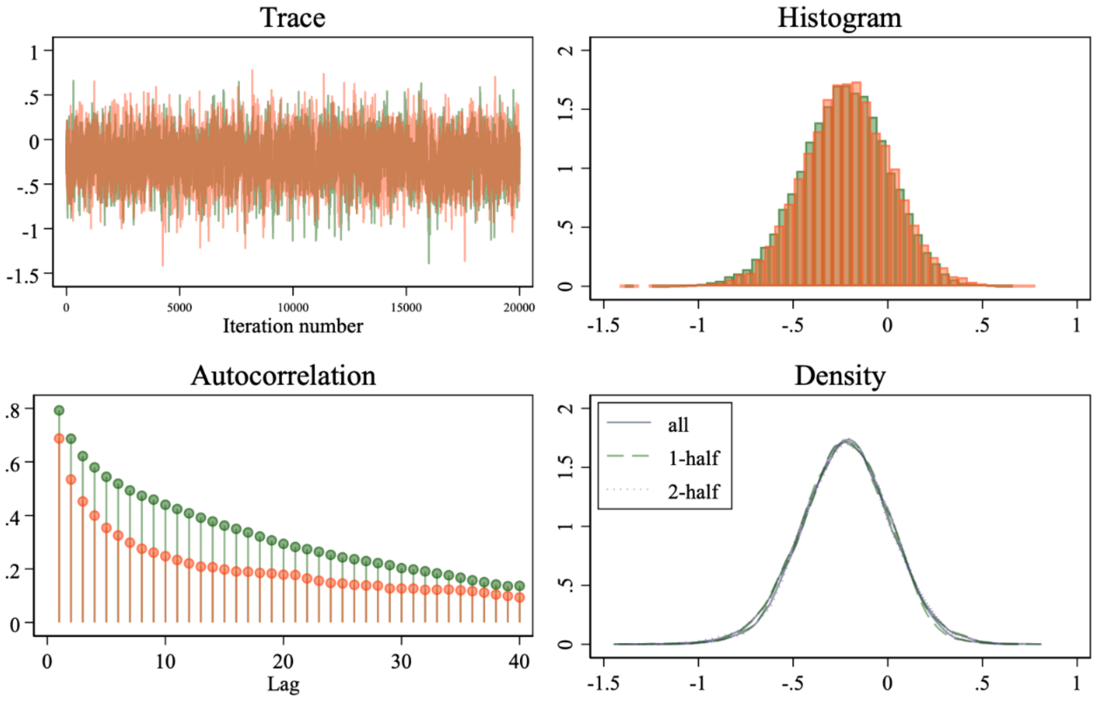

Supplementary Figure 15. Convergence Criteria for Overconfidence; Age Parameter

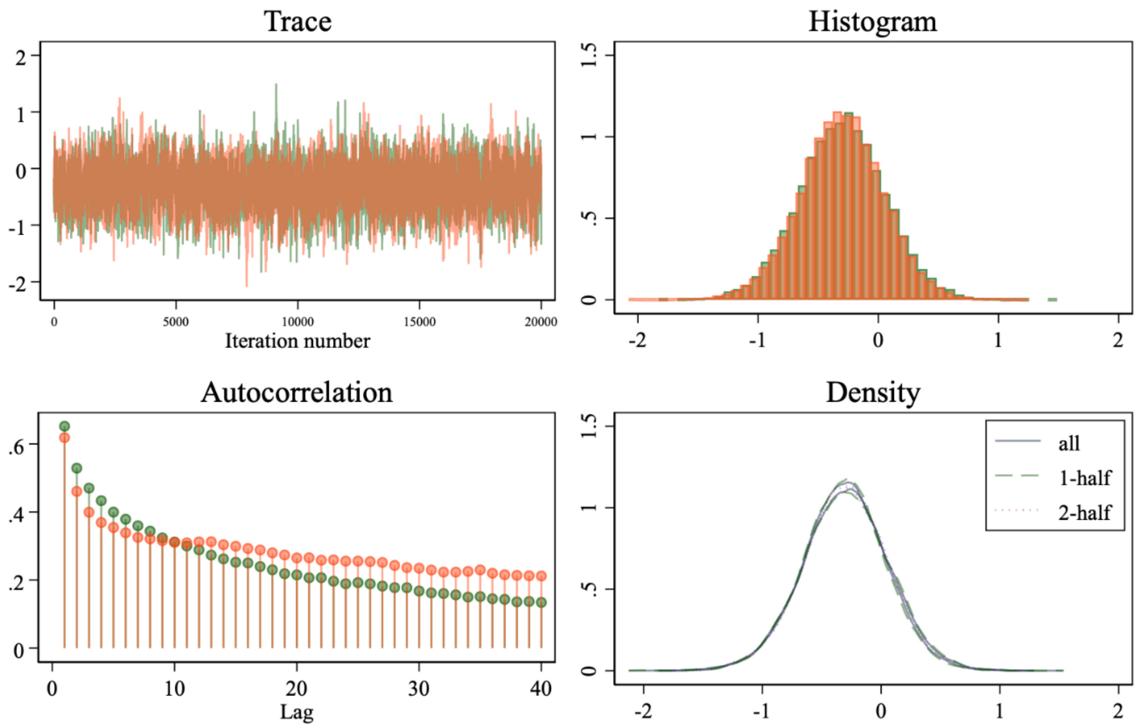

Supplementary Figure 16. Convergence Criteria for Overconfidence; Gender Parameter

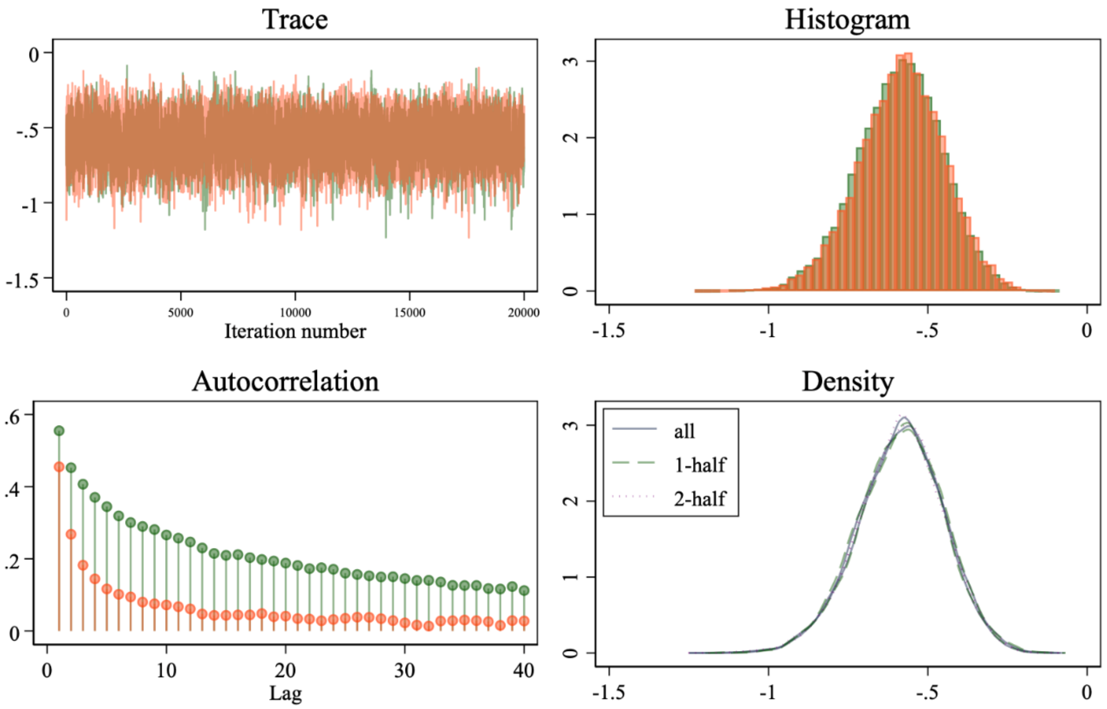

Supplementary Figure 17. Convergence Criteria for Overconfidence; Time Parameter

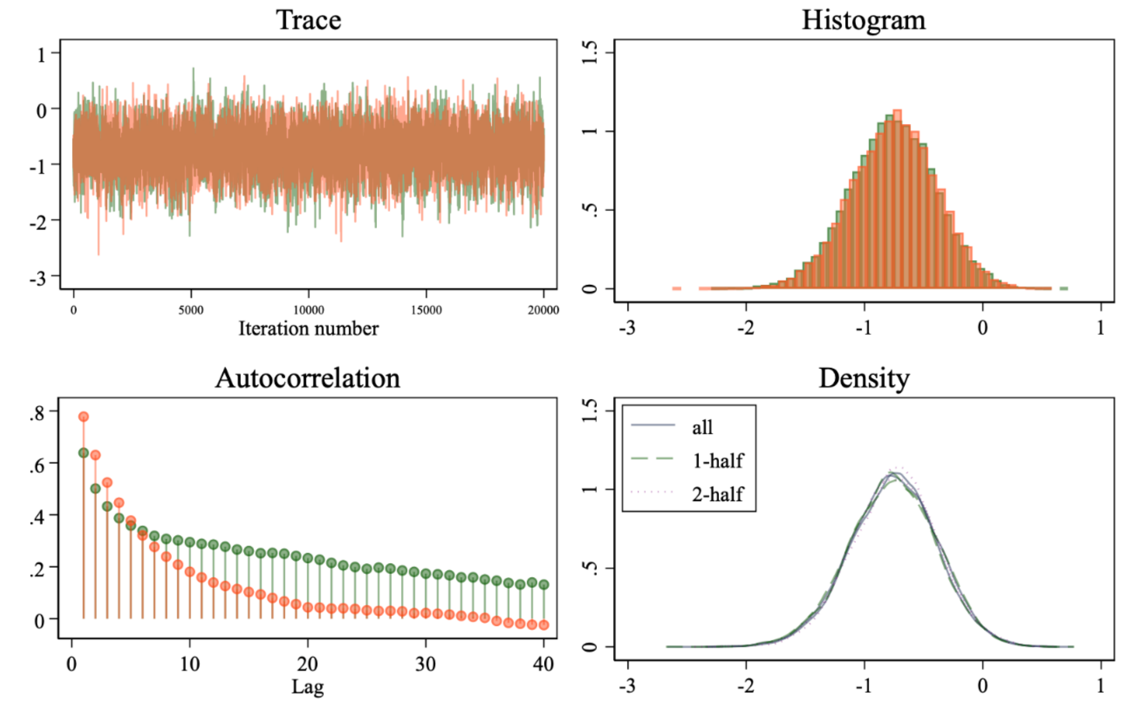

Supplementary Figure 18. Convergence Criteria for Overconfidence; Interaction (Gender x 2.Estimate) Parameter

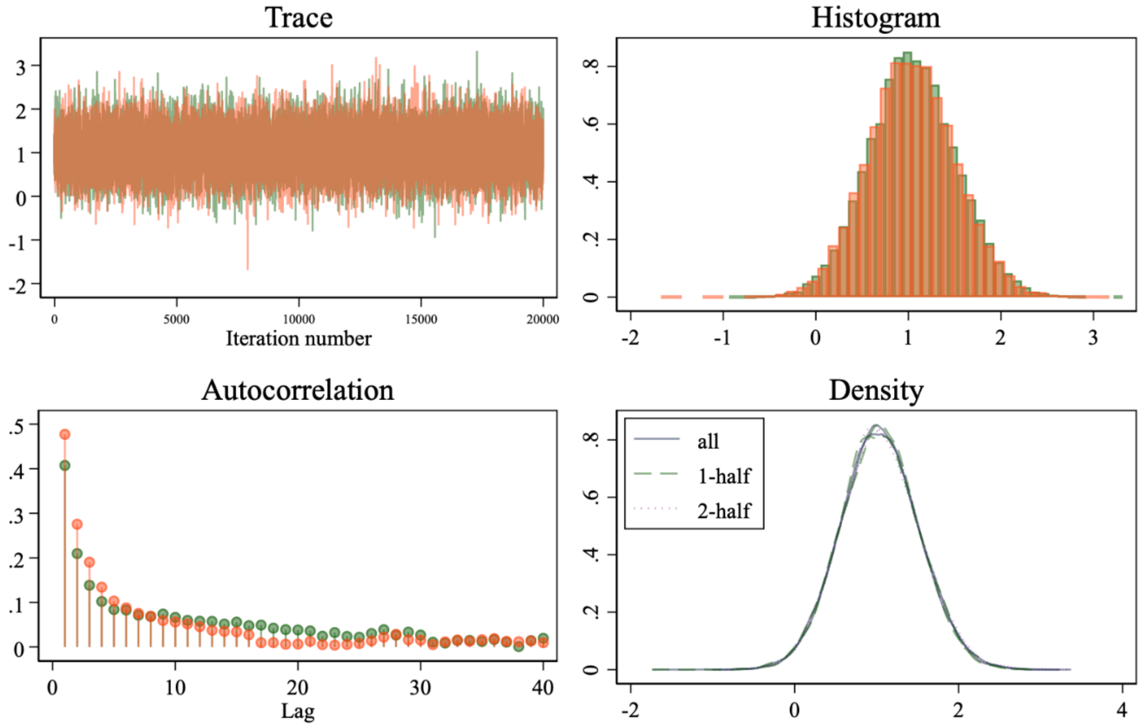

Supplementary Figure 19. Convergence Criteria for Overconfidence; Interaction (Gender x 4. Estimate) Parameter

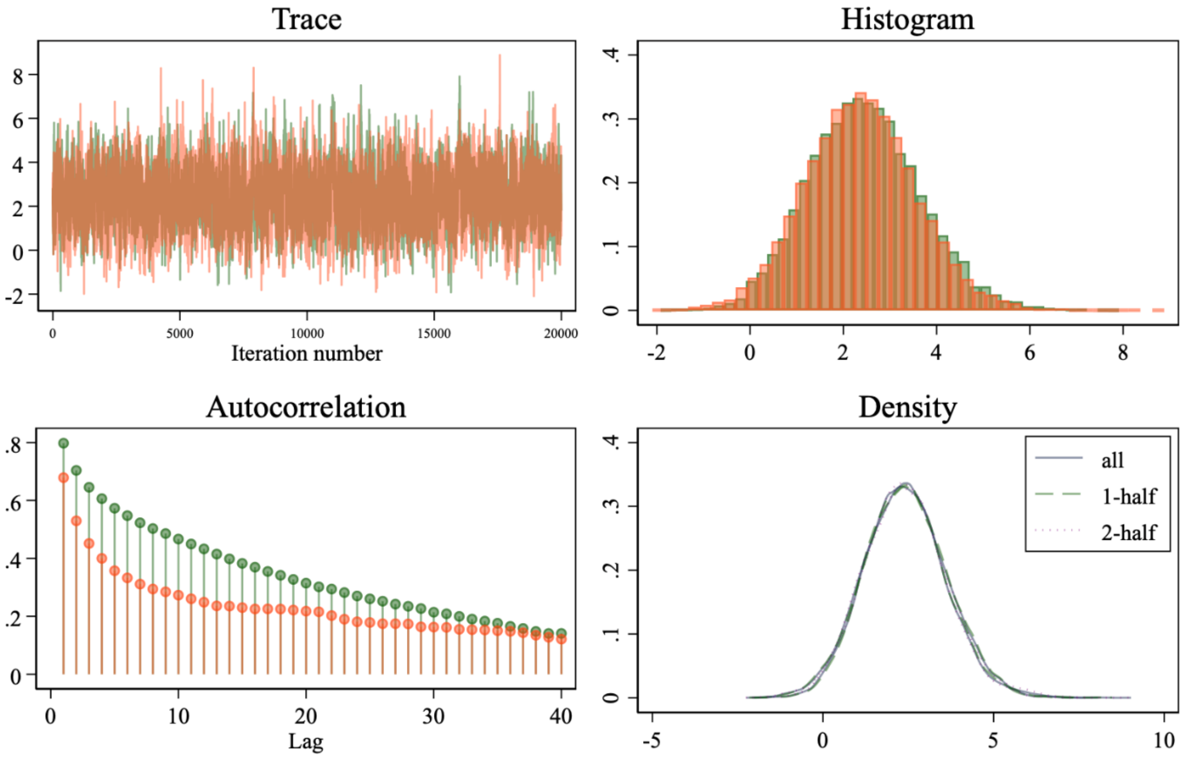

Supplementary Figure 20. Convergence Criteria for Overconfidence; Constant Parameter

Robustness Check: Miscalibration

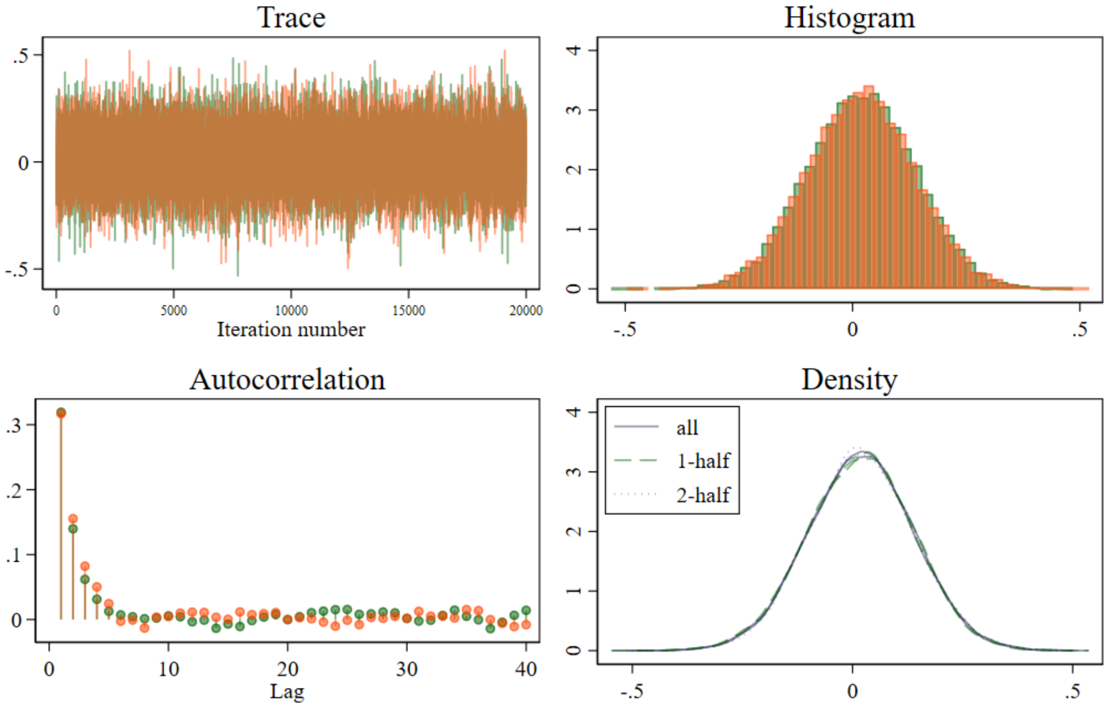

Supplementary Figure 21. Convergence Criteria for Miscalibration; Intervention Parameter

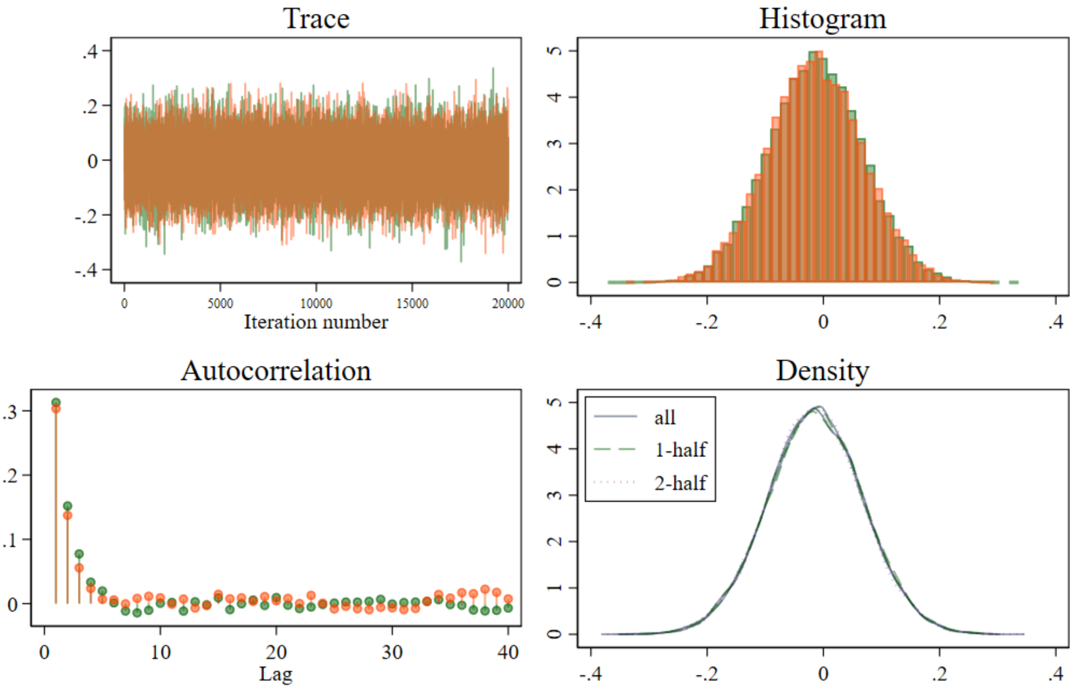

Supplementary Figure 22. Convergence Criteria for Miscalibration; Age Parameter

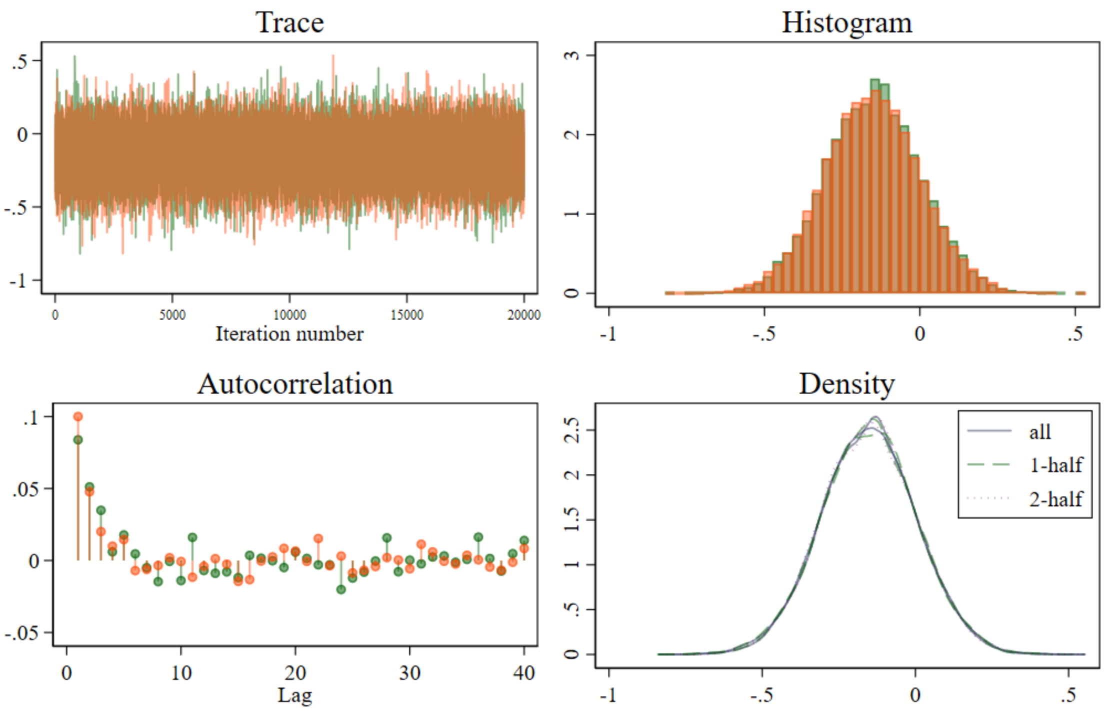

Supplementary Figure 23. Convergence Criteria for Miscalibration; Gender Parameter

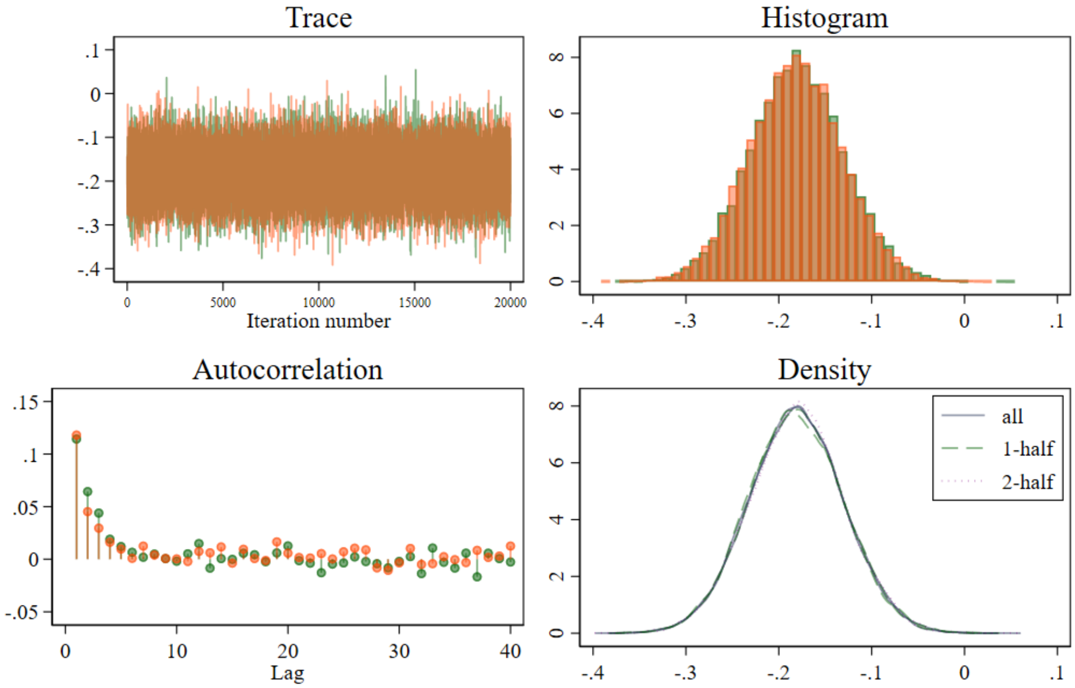

Supplementary Figure 24. Convergence Criteria for Miscalibration; Time Parameter

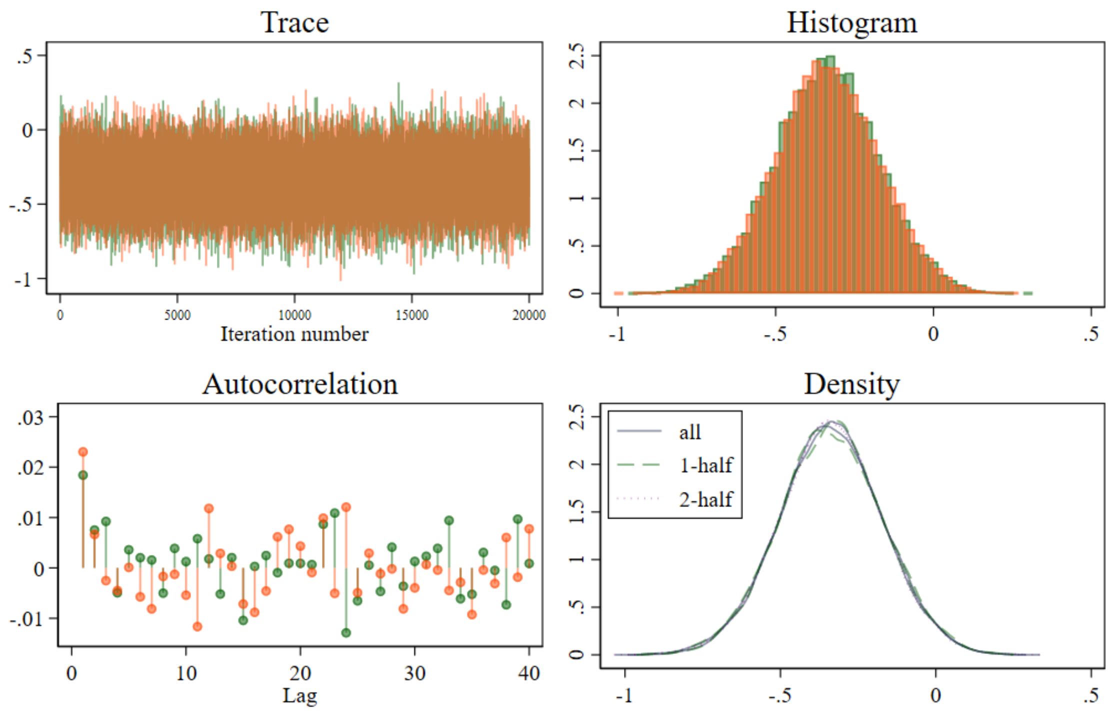

Supplementary Figure 25. Convergence Criteria for Miscalibration; Interaction (Gender x 2.Estimate) Parameter

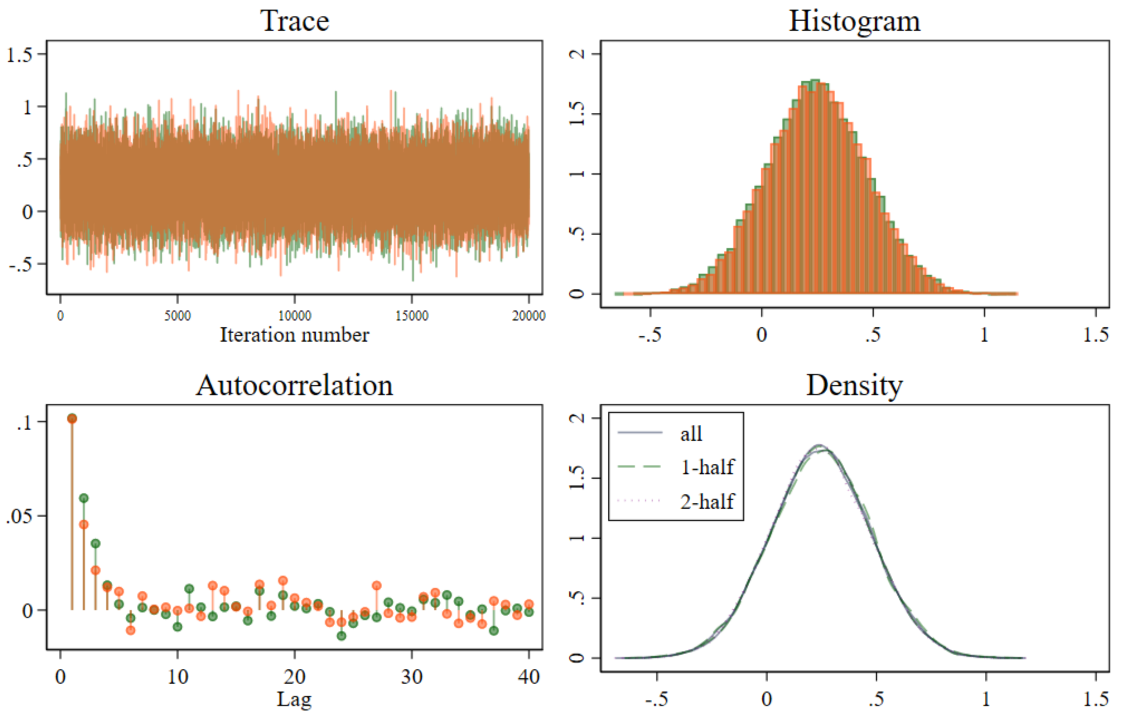

Supplementary Figure 26. Convergence Criteria for Miscalibration; Interaction (Gender x 4.Estimate) Parameter

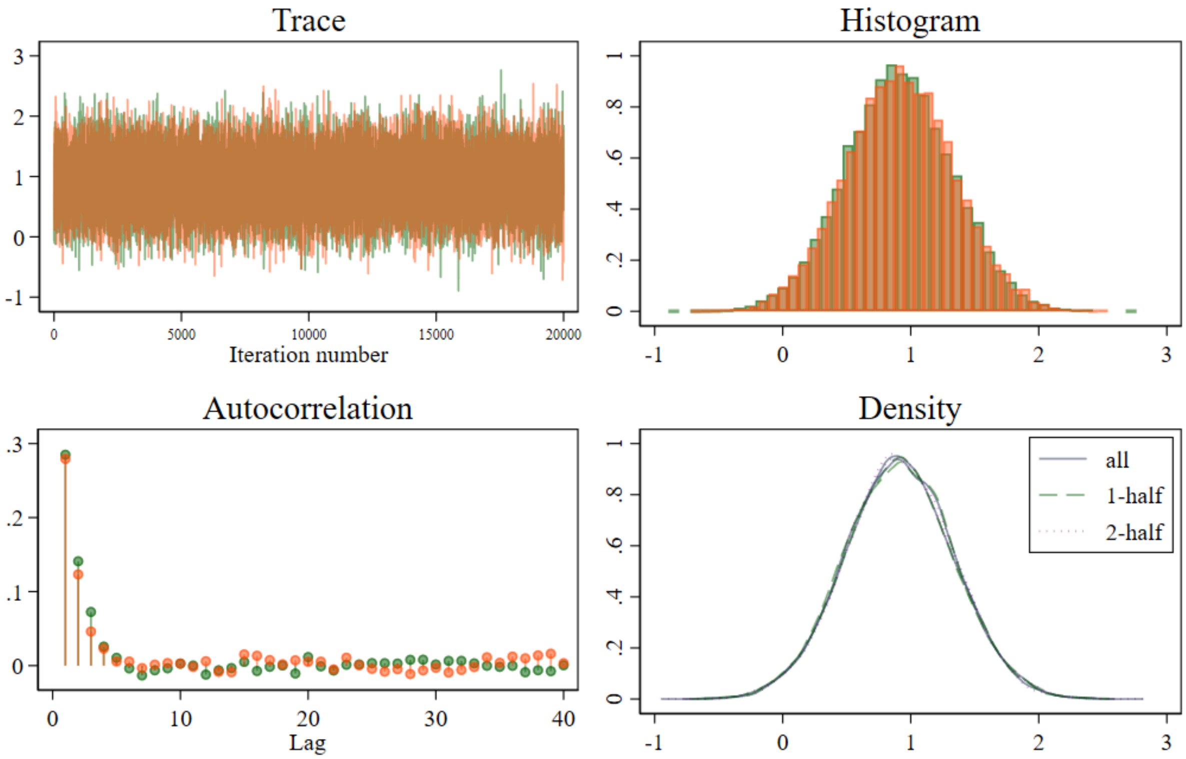

Supplementary Figure 27. Convergence Criteria for Miscalibration; Constant Parameter
